# Supplementary material for: PhyloMix: enhancing microbiome-trait association prediction through phylogeny-mixing augmentation
Source: Bioinformatics. 2025 Jan 12;41(2):btaf014. doi: 10.1093/bioinformatics/btaf014 (PMC11849959; doi:10.1093/bioinformatics/btaf014)
Supplement: btaf014_Supplementary_Data [file btaf014_supplementary_data.zip › phylomix_supp.pdf]

Supplement to "PhyloMix: Enhancing microbiome-trait association prediction through phylogeny-mixing augmentation"

Yifan Jiang[1,*], Disen Liao[1,*], Qiyun Zhu[2], and Yang Young Lu*[1]

[1]Cheriton School of Computer Science, University of Waterloo, Waterloo, Ontario, Canada
[2]School of Life Sciences, Arizona State University, Tempe, Arizona, USA

S1 The rationale of data augmentation

Our analysis demonstrates that across all datasets, mixup-based data augmentation methods–including the vanilla mixup [Zhang et al., 2018], the compositional cutmix [Gordon-Rodriguez et al., 2022], and PhyloMix–consistently outperform generative data augmentation approaches such as TADA [Sayyari et al., 2019] and MB-GAN [Rong et al., 2021]. Beyond the empirical results, mixup-based data augmentation methods are theoretically analogous to certain regularization techniques, such as label smoothing and dropout [Carratino et al., 2022]. This is due to their inherent ability to pull both inputs and outputs closer to their mean, which enhances model calibration and smooths the model's Jacobian, ultimately improving generalization.

To illustrate the effect of data augmentation, we visualize how it smooths the decision boundary of the SVM model. Specifically, we trained an SVM model on the training split of the IBD dataset, calculated the decision boundary, and examined how this boundary separates positive and negative samples in the test split. As shown in Fig. S1, after data augmentation, the decision boundary shifts toward the mean of the output space, leading to a smoother and more generalized boundary. Note that the data augmentation is particularly effective at smoothing the decision boundary when the sample size is small.

*Correspondence: yanglu@uwaterloo.ca

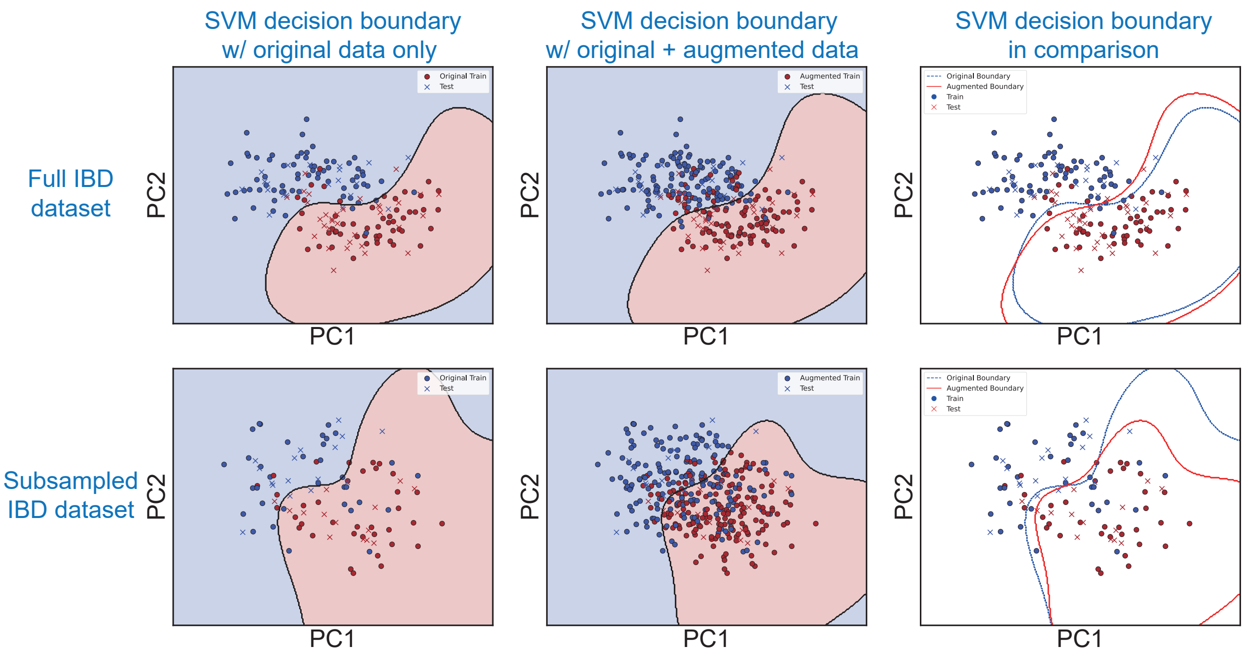

Figure S1: **PhyloMix smooths the decision boundary of the SVM model.** The evaluation was performed on the IBD dataset using both the full dataset and a subsampled version. Data augmentation is particularly effective at smoothing the decision boundary when the sample size is small.

S2 Dataset details

PhyloMix used six publicly available microbiome datasets with varying sample sizes and feature dimensionality, with details listed in Tab. S1.

S2.1 Simulated datasets

Additionally, we created three simulated datasets using the microbiome data simulator MIDASim [He et al., 2024]. MIDASim generates simulated data by leveraging a template microbiome dataset and preserving its correlation structure to ensure similarity. Specifically, we employed the parametric mode of MIDASim, utilizing a generalized gamma distribution to model the relative abundances of microbiome data. This approach is tailored for simulation studies that involve modifying the log-mean relative abundance. Since MIDASim is not designed to generate simulated samples with labels, we selected a real dataset with positive and negative labels. We then simulated samples separately for each label (positive and negative) before combining them into a unified simulated dataset. The real dataset we chose is the IBD dataset [Gonzalez et al., 2022] studied the relationship between the gut microbiome and two main subtypes of inflammatory bowel disease (IBD): Crohn's disease (CD) and ulcerative colitis (UC). It includes 108 CD and 66 UC samples, with profiles containing $p = 5287$ taxa.

We simulated three distinct datasets from the IBD dataset. We estimated separate location parameters for each of the two labels from the IBD dataset, denoted as $\mu_+ \in \mathbb{R}^{5287}$ and $\mu_- \in \mathbb{R}^{5287}$. After that, we varied the location parameters to represent different levels of difficulty in distinguishing between labels, as follows:

- Setting 1: $(2 * \mu_+)$ and $(2 * \mu_-)$. (Alg. 1)
- Setting 2: $(\mu_+ + 5)$ and $(\mu_- + 5)$. (Alg. 2)
- Setting 3: Randomly select 10% of taxa with non-zero abundance and increase their values by 10%. (Alg. 3)

Three settings introduce progressively greater challenges. Setting 1 involves minor deviations that preserve the presence-absence pattern but introduce moderate variability in relative abundances. In this setting, we doubled the

Table S1: The details of the real datasets investigated by PhyloMix.

Dataset	Sample size	Feature size	Data source	Notes
AlzBiom	175 samples (75 amyloid-positive and 100 healthy control)	8,350 taxa	EBI-ENA ID: PRJEB47976	The sequencing data is clean (QC'ed, host-filtered).
ASD	60 samples (30 typically developing and 30 constipated ASD)	7,287 taxa	EBI-ENA ID: PRJNA451479	The sequencing data is raw (non-QC'ed).
GD	162 samples (100 Graves' disease and 62 healthy control)	8,487 taxa	EBI-ENA ID: PRJNA602729, PRJNA602731, PRJNA602732, PRJNA638403, PRJNA638404, PRJNA638405	Most samples have a pair of FASTQ files. However, 4 samples (three.lst) have a third, unpaired FASTQ file that is very small, and it should be excluded from the analysis. 12 samples have only one FASTQ file, which appears to be single-end sequences. Two samples: GA61 (SRR12000211) and GA89 (SRR12005695) are missing from the metadata. Therefore they were dropped from the data.
RUMC	114 samples (42 Parkinson's disease and 72 healthy control)	7,256 taxa	Qiita ID: 12975	20 samples in BIOM are missing in metadata. These samples were dropped.
IBD	174 samples (108 Crohn's disease and 66 ulcerative colitis)	5,287 taxa	Qiita ID: 12675	The dataset contains metagenomic sequencing data and associated metadata. More details can be found at: `https://qiita.ucsd.edu/study/description/12675`
HMP2	1,158 samples (728 Crohn's disease and 430 ulcerative colitis)	10,614 taxa	Qiita ID: 11484	The dataset contains metagenomic sequencing data and associated metadata from the Human Microbiome Project. More details can be found at: `https://hmpdacc.org/ihmp`

mean location parameters of the gamma distribution for both positive and negative samples. This transformation preserves the presence-absence status of the taxa but shifts the location of the gamma distribution for each taxon. As a result, the relative abundance of taxa is altered due to the inherent randomness of the gamma distribution, though these changes remain relatively modest. Setting 2 significant shifts in distribution occur here, altering the relative abundance patterns substantially. In this setting, we shifted the mean location parameters by 5 units for both positive and negative samples. This adjustment ensures the presence of all taxa in the simulated dataset while significantly altering the underlying distribution. Consequently, the dataset exhibits greater deviations in relative abundance compared to the template, thereby increasing its complexity. In Setting 3, the original distribution structure is disrupted by directly modifying a subset of taxa, leading to pronounced deviations that may obscure the underlying data patterns. In this setting, the manipulation leads to substantial deviations from the original template. By altering the relative abundance of a subset of taxa, the overall structure of the dataset is disrupted, making this the most challenging scenario for model learning and inference. For each simulated dataset, we generated 100 positive samples and 100 negative samples.

Algorithm 1 Simulation Setting 1: Doubling the Location Parameter

Require: Fitted gamma model parameters μ_j^+, μ_j^-, and library sizes N_i^+, N_i^-.

1: Update the location parameters:

$$\mu_j^+ \leftarrow 2\mu_j^+, \quad \mu_j^- \leftarrow 2\mu_j^-.$$

2: Sample survival times $\tilde{t}_{ij}^+$ and $\tilde{t}_{ij}^-$ from the updated generalized gamma distributions truncated by N_i^+ and N_i^-, respectively.

3: Compute relative abundances by inverting survival times:

$$\tilde{\pi}_{ij}^+ = \frac{1}{\tilde{t}_{ij}^+}, \quad \tilde{\pi}_{ij}^- = \frac{1}{\tilde{t}_{ij}^-}.$$

4: Compute sumulated counts using the library sizes:

$$\tilde{C}_{ij}^+ = \tilde{\pi}_{ij}^+ \cdot sN_i^+, \quad \tilde{C}_{ij}^- = \tilde{\pi}_{ij}^- \cdot N_i^-.$$

5: **Output:** Simulated dataset where taxa presence-absence status is preserved but relative abundances exhibit moderate changes.

Algorithm 2 Simulation Setting 2: Shifting the Location Parameter

Require: Fitted gamma model parameters μ_j^+, μ_j^-, and library sizes N_i^+, N_i^-.

1: Update the location parameters:

$$\mu_j^+ \leftarrow \mu_j^+ + 5, \quad \mu_j^- \leftarrow \mu_j^- + 5.$$

2: Sample survival times $\tilde{t}_{ij}^+$ and $\tilde{t}_{ij}^-$ from the updated generalized gamma distributions truncated by N_i^+ and N_i^-, respectively.

3: Compute relative abundances by inverting survival times:

$$\tilde{\pi}_{ij}^+ = \frac{1}{\tilde{t}_{ij}^+}, \quad \tilde{\pi}_{ij}^- = \frac{1}{\tilde{t}_{ij}^-}.$$

4: Compute sumulated counts using the library sizes:

$$\tilde{C}_{ij}^+ = \tilde{\pi}_{ij}^+ \cdot sN_i^+, \quad \tilde{C}_{ij}^- = \tilde{\pi}_{ij}^- \cdot N_i^-.$$

5: **Output:** Simulated dataset where taxa presence-absence status is preserved but relative abundances exhibit moderate changes.

Algorithm 3 Simulation Setting 3: Modifying Relative Abundance

Require: Simulated relative abundances π_{ij}, and library sizes N_i^+, N_i^-.

1: Randomly select 10% of taxa from both positive and negative samples.

2: **for** each selected taxon j **do**

3: Compute relative abundances by inverting survival times and increasing non-zero relative abundances by 10%:

$$\pi_{ij}^+ \leftarrow \pi_{ij}^+ \cdot 1.1, \quad \pi_{ij}^- \leftarrow \pi_{ij}^- \cdot 1.1, \quad \text{if } \pi_{ij} > 0.$$

4: **end for**

5: Compute simulated counts counts using new relative abundance and library sizes:

$$\tilde{C}_{ij}^+ = \pi_{ij}^+ \cdot N_i^+, \quad \tilde{C}_{ij}^- = \pi_{ij}^- \cdot N_i^-, \quad \forall i, j$$

6: **Output:** Simulated dataset with significantly altered relative abundance patterns and higher complexity.

S3 Benchmark details

PhyloMix is used together with five ML models with varying predictive capabilities: logistic regression (LR), support vector machine (SVM) with a linear kernel, random forest (RF), multi-layer perceptron (MLP), and MIOSTONE, a state-of-the-art deep learning model that encodes taxonomy [Jiang et al., 2023]. We used the Scikit-learn implementation [Pedregosa et al., 2011] with default settings for the LR, RF, and SVM models. Specifically, the logistic regression is trained using L2 regularization with a coefficient of 1.0. The random forest classifier is trained with 100 trees, without a maximum tree depth constraint, and a minimum of 2 samples required to split an internal node. The support vector classifier is trained with a linear kernel, using L2 regularization with a coefficient of 1.0. The MLP model was configured with a pyramid-shaped architecture featuring two hidden layers of size 256 and 128, respectively. For the MIOSTONE model, we employed the default implementation settings. We trained both the MLP and MIOSTONE models for 200 epochs with a batch size of 512 to ensure convergence. During training, we used the AdamW optimizer with a learning rate of 0.001 and applied a cosine annealing scheduler. It is important to note that we used the same model settings to train both the original and augmented data, ensuring a fair comparison.

For all methods, we preprocessed microbiome features using centered log-ratio transformation (CLR) [Aitchison, 1982] prior to data augmentation. To showcase the broad applicability of PhyloMix, we also conducted experiments using relative abundance data, which was obtained by normalizing the original count data without applying a centered log-ratio transformation. In this setup, we replaced the linear kernel in the SVM with a Radial Basis Function (RBF) kernel to better capture the characteristics of the data.

S4 Polytomy resolution

PhyloMix uses the Web of Life (WoL) phylogeny [Zhu et al., 2019], which includes $15,953$ microbial genomes. Note that a small number of internal nodes in the WoL phylogeny have more than two lineages (*i.e.,* polytomies). The process of resolving polytomies, *i.e.,* converting them into bifurcating phylogenies, can be either stochastic or deterministic. The stochastic approach arbitrarily assigns a dichotomous structure to nodes with more than two lineages, while the deterministic approach systematically creates a dichotomous structure, beginning with the leftmost child node. As shown in Fig. S2, the performance of PhyloMix remains robust regardless of the polytomy resolution strategy applied to the phylogeny.

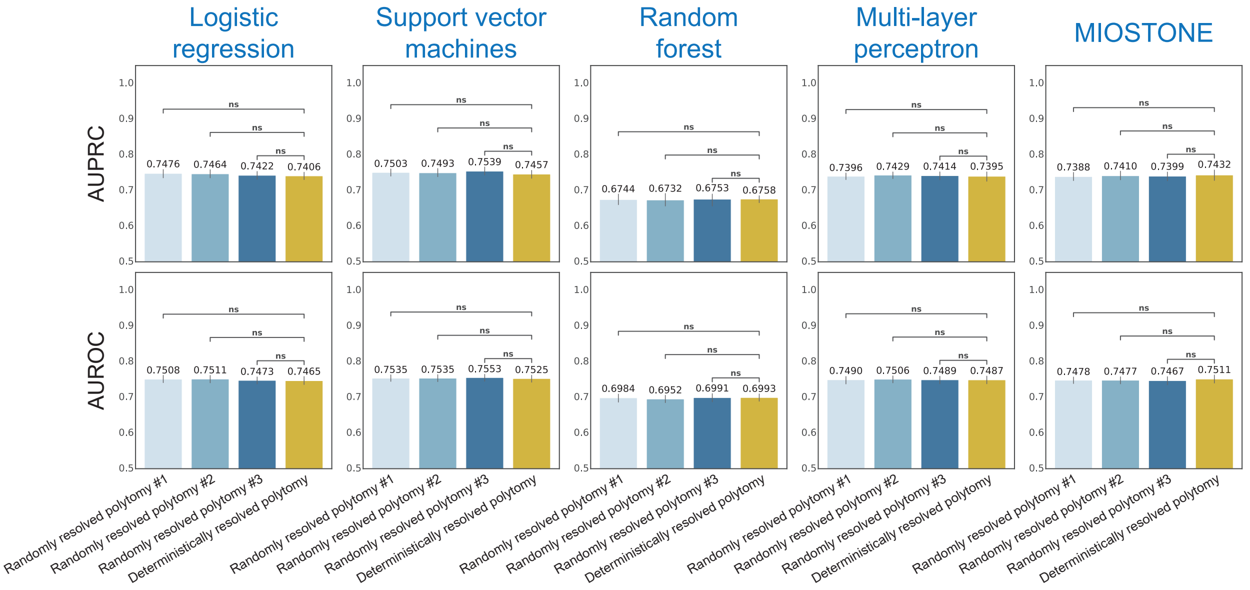

Figure S2: **PhyloMix's performance remains robust regardless of the polytomy resolution strategy applied to the phylogeny.** The evaluation was conducted on the IBD dataset and compared against five distinct baseline methods. The WoL phylogeny contains a small number of internal nodes having more than two lineages (*i.e.,* polytomies), which is resolved using either stochastic or deterministic. To ensure scientific rigor, the stochastic polytomy resolution is performed three times using different random seeds. PhyloMix with different polytomy resolution strategies are measured by both AUPRC and AUROC.

S5 Alpha and beta diversity preservation

We calculated the alpha and beta diversity of both the original and PhyloMix-augmented data across six publicly available microbiome datasets. This approach aimed to evaluate whether the augmented data accurately captured the overall correlation structure patterns of the original data. We used the Shannon Index to measure alpha diversity and the Bray-Curtis metric for beta diversity. As shown in Fig. S3, PhyloMix's augmented data preserves both the alpha diversity and beta diversity of the original data across six real datasets. We calculated the p-value for alpha diversity using the Kruskal-Wallis test and for beta diversity using PERMANOVA, with the results presented in Tab. S2.

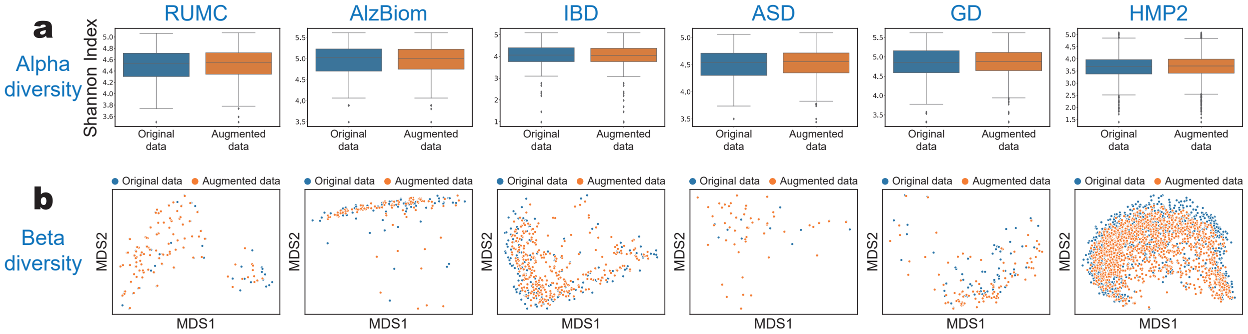

Figure S3: **PhyloMix's augmented data preserves both the alpha diversity and beta diversity of the original data.** (**a**) The box plots of the alpha diversity across six real datasets, calculated for each sample in the original data and the augmented data generated by PhyloMix. (**b**) Beta diversity is calculated for every pair of samples from either the original data or the augmented data. The resulting pairwise diversity matrix is visualized in a 2D space using multidimensional scaling (MDS).

Table S2: P-values for alpha diversity and beta diversity between the original data and PhyloMix's augmented data.

Diversity Metric	IBD	AlzBiom	ASD	RUMC	GD	HMP2
Shannon Index	0.7105	0.8126	0.9106	0.5904	0.7288	0.2126
Bray-Curtis	0.9980	1.0000	1.0000	0.9970	1.0000	1.0000

S6 Supervised learning results

S6.1 Data with centered log-ratio transformation

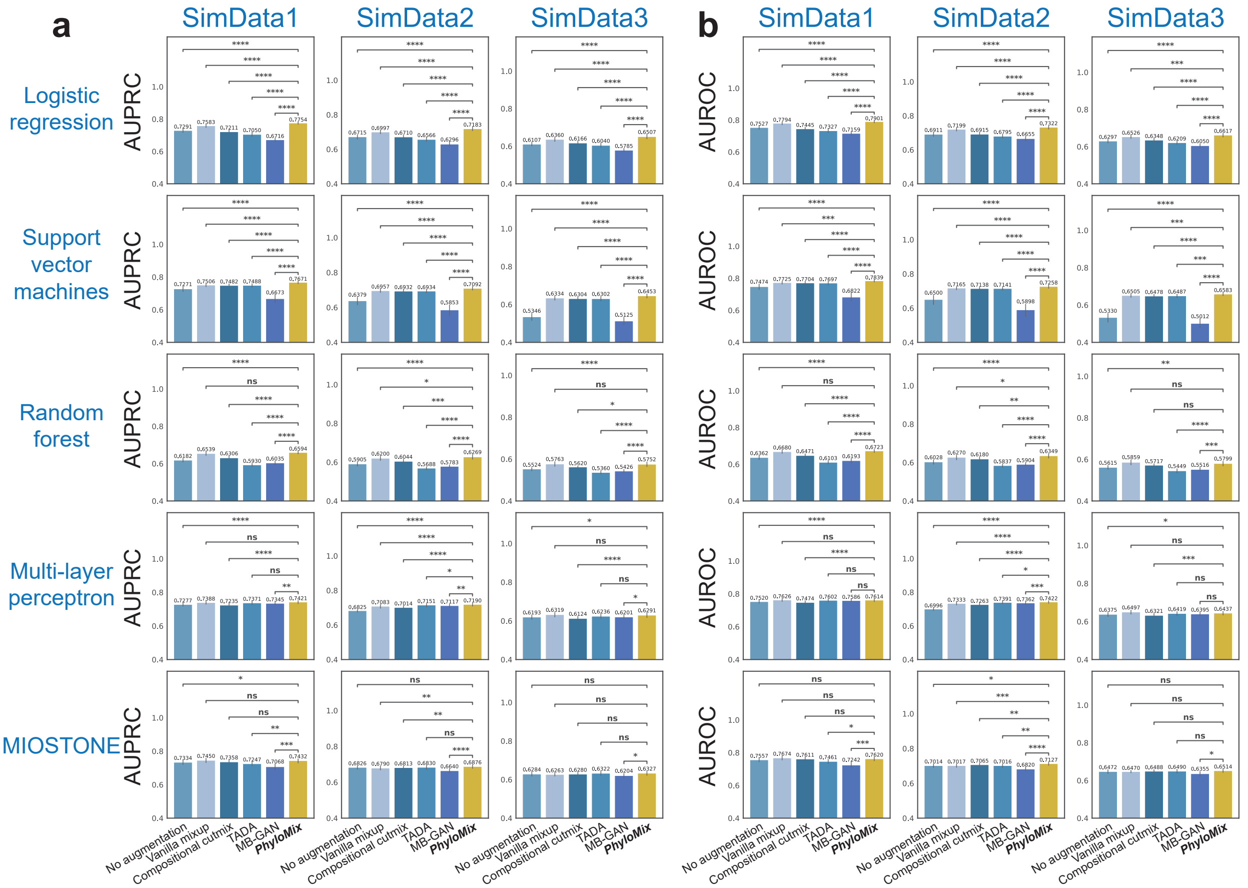

Figure S4: **Data augmentation performance on the three simulated datasets in the supervised learning setting.** PhyloMix is evaluated alongside five ML models with varying predictive capabilities and compared against four distinct baseline methods. We preprocessed microbiome features using centered log-ratio transformation prior to data augmentation. The performance is measured by (**a**) AUPRC and (**b**) AUROC. For scientific rigor, the performance comparison between PhyloMix and other baseline methods is quantified using one-tailed two-sample t-tests to calculate p-values: $****$: p-value ≤ 0.0001; $***$: p-value ≤ 0.001; $**$: p-value ≤ 0.01; $*$: p-value ≤ 0.05; ns : p-value > 0.05.

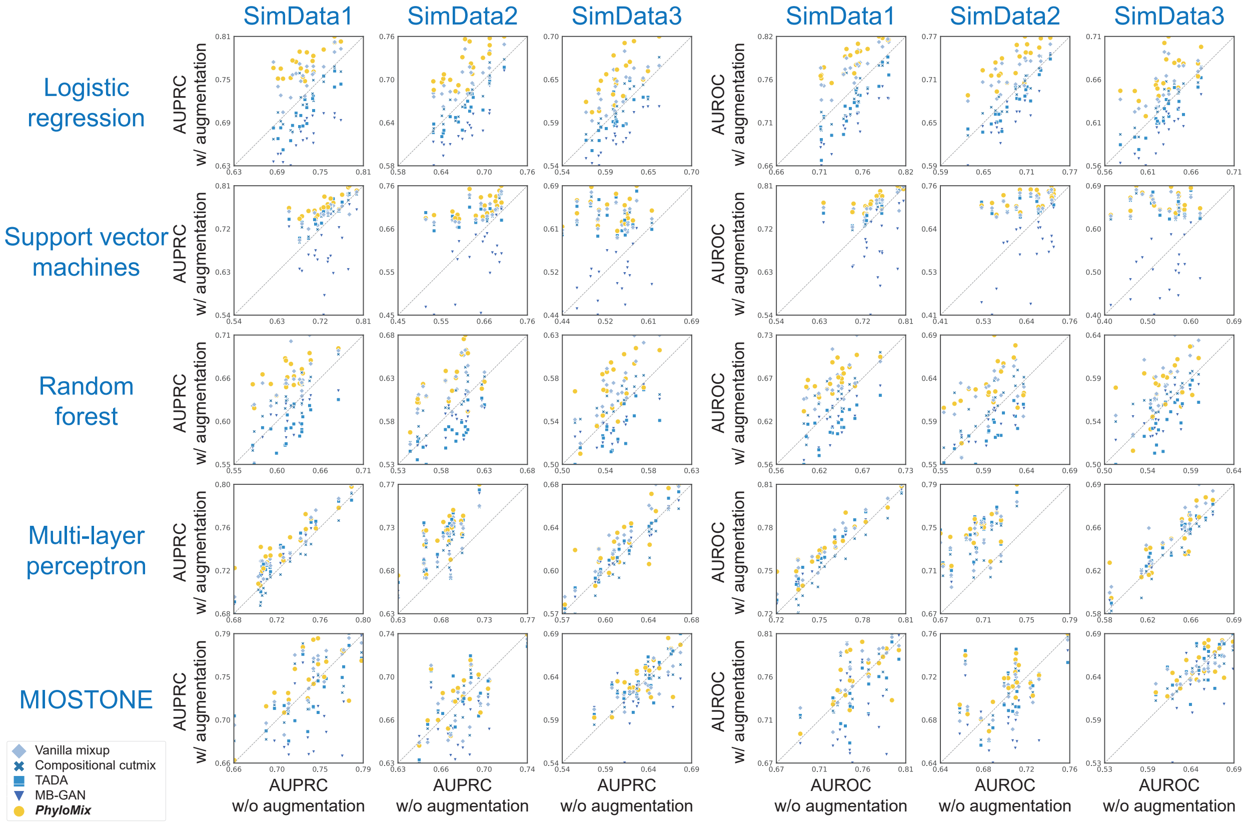

Figure S5: **Qualitative evaluation of PhyloMix's performance on the three simulated datasets in the supervised learning setting.** Each dot corresponds to the performance with or without data augmentation for a given method under a random seed. The performance is measured by (**a**) AUPRC and (**b**) AUROC.

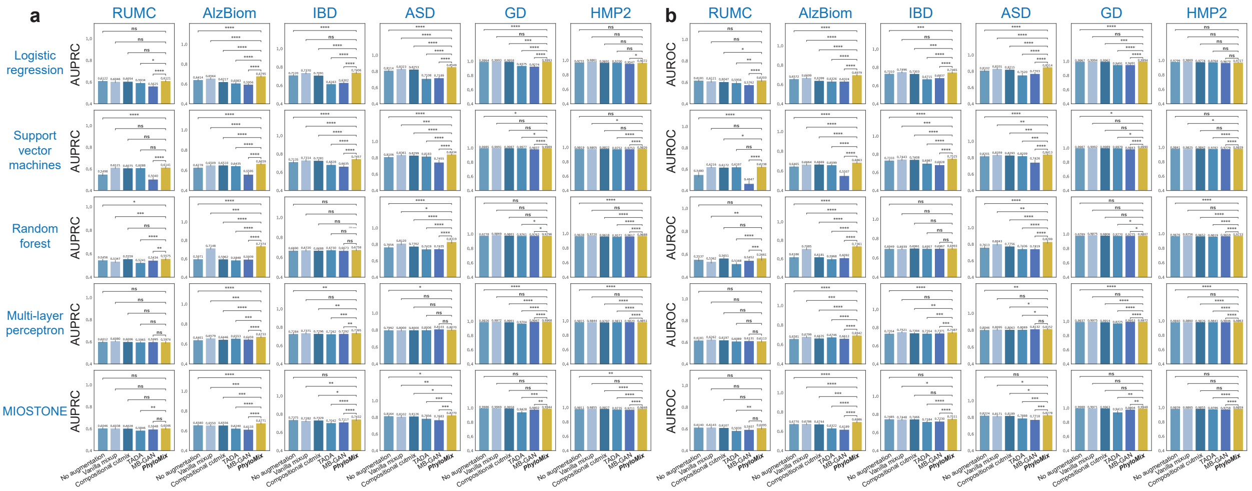

Figure S6: **Data augmentation performance on the six real datasets in the supervised learning setting.** PhyloMix is evaluated alongside five ML models with varying predictive capabilities and compared against four distinct baseline methods. We preprocessed microbiome features using centered log-ratio transformation prior to data augmentation. The performance is measured by (**a**) AUPRC and (**b**) AUROC. For scientific rigor, the performance comparison between PhyloMix and other baseline methods is quantified using one-tailed two-sample t-tests to calculate p-values: $****$: p-value ≤ 0.0001; $***$: p-value ≤ 0.001; $**$: p-value ≤ 0.01; $*$: p-value ≤ 0.05; ns : p-value > 0.05.

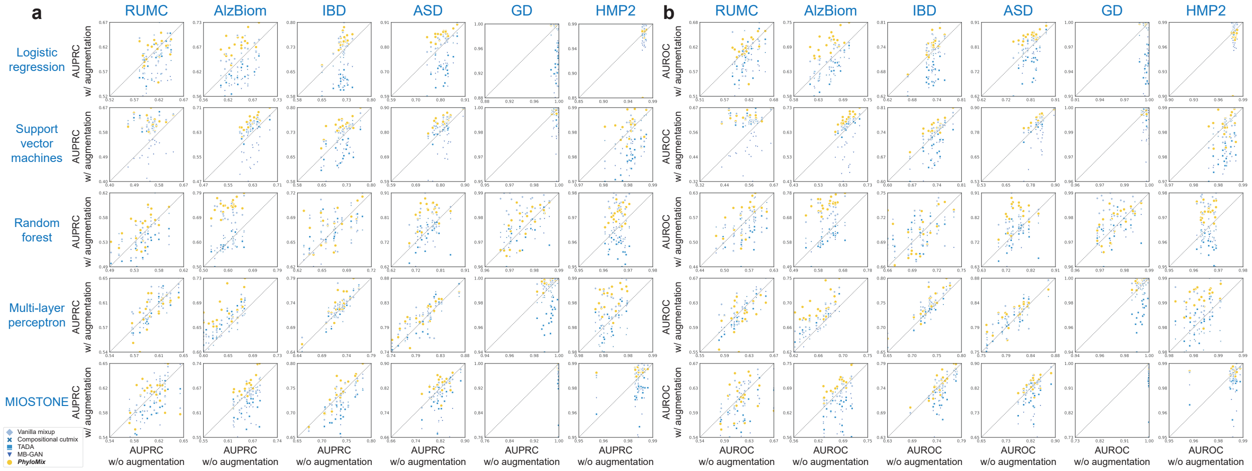

Figure S7: **Qualitative evaluation of PhyloMix's performance on the six real datasets in the supervised learning setting.** Each dot corresponds to the performance with or without data augmentation for a given method under a random seed. The performance is measured by (**a**) AUPRC and (**b**) AUROC.

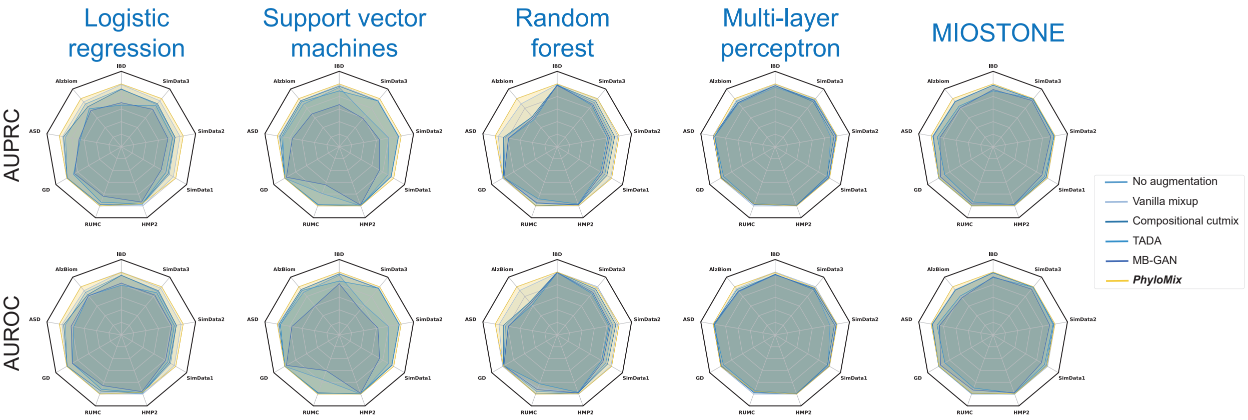

Figure S8: **Comparing PhyloMix against baselines methods on all datasets in the supervised learning setting using radar plots.** Performance is evaluated using relative AUPRC or AUROC, calculated by normalizing the values against the best performer across all methods.

S6.2 Data with relative abundance normalization

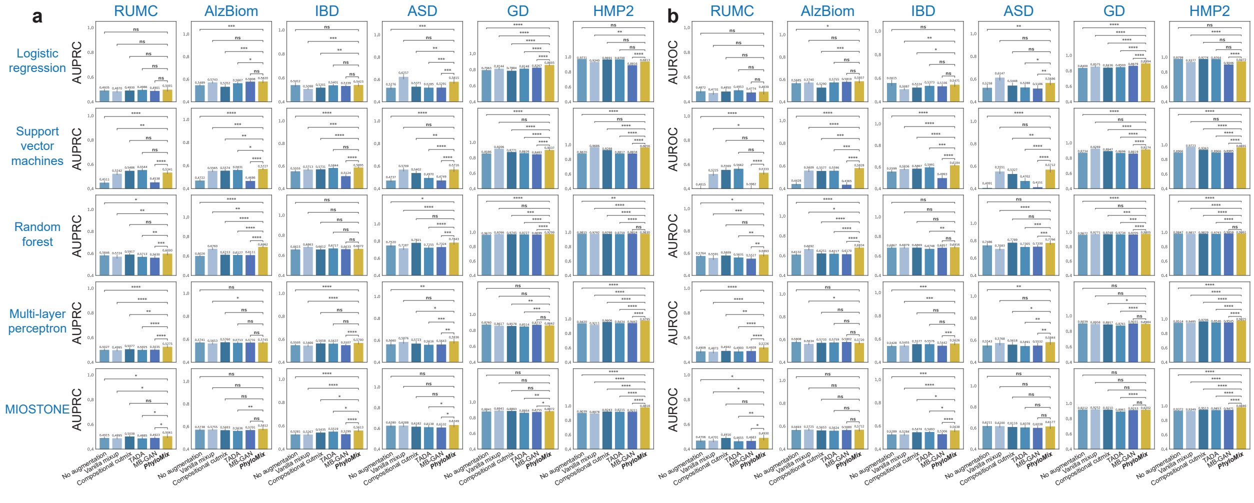

Figure S9: **Data augmentation performance on the six real datasets in the supervised learning setting.** PhyloMix is evaluated alongside five ML models with varying predictive capabilities and compared against four distinct baseline methods. We preprocessed microbiome features using relative abundance normalization prior to data augmentation. The performance is measured by (**a**) AUPRC and (**b**) AUROC. For scientific rigor, the performance comparison between PhyloMix and other baseline methods is quantified using one-tailed two-sample t-tests to calculate p-values: $****$: p-value ≤ 0.0001; $***$: p-value ≤ 0.001; $**$: p-value ≤ 0.01; $*$: p-value ≤ 0.05; ns : p-value > 0.05.

S7 Ablation results

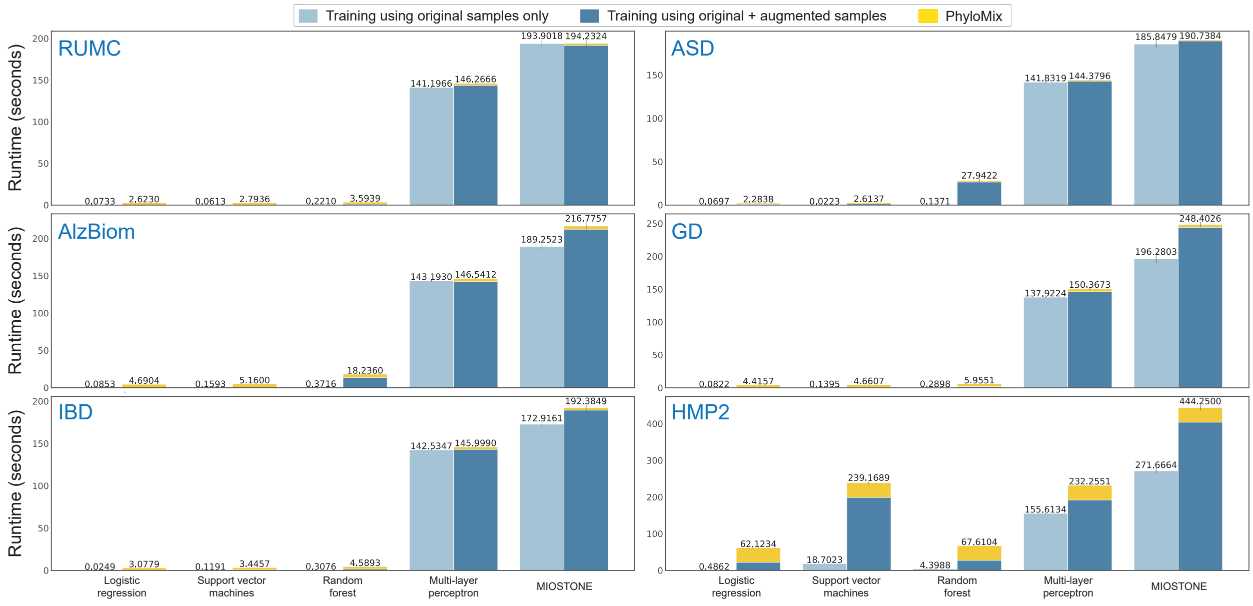

Figure S10: **The computational cost of PhyloMix.**

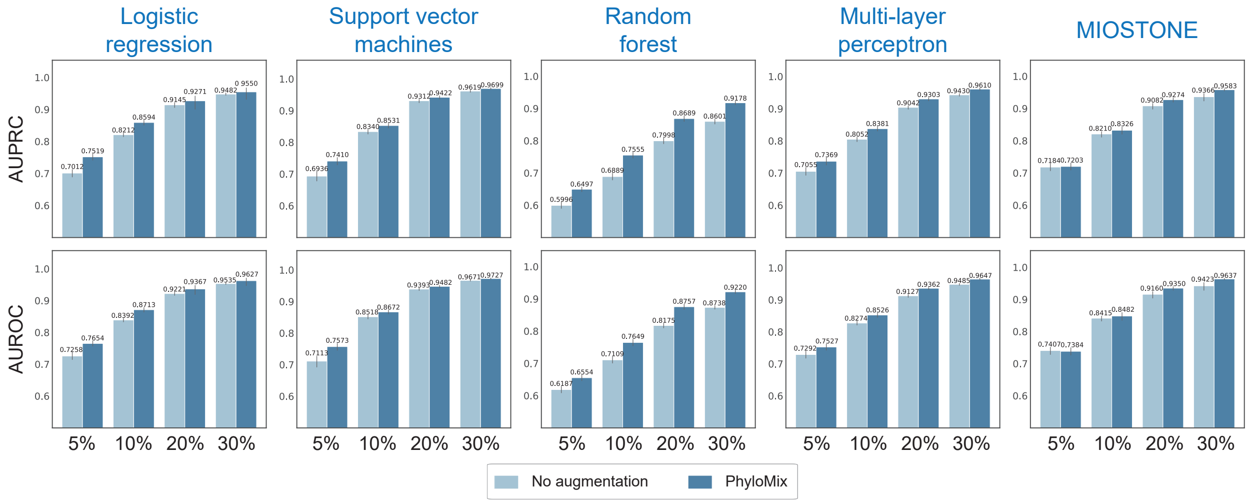

Figure S11: **PhyloMix exhibits more significant improvements when the training data size is small.**

References

J. Aitchison. The statistical analysis of compositional data. Journal of the Royal Statistical Society: Series B (Methodological), 44(2):139–160, 1982.

L. Carratino, M. Cissé, R. Jenatton, and J.-P. Vert. On mixup regularization. Journal of Machine Learning Research, 23(325):1–31, 2022.

C. G. Gonzalez, R. H. Mills, Q. Zhu, C. Sauceda, R. Knight, P. S. Dulai, and D. J. Gonzalez. Location-specific signatures of Crohn's disease at a multi-omics scale. Microbiome, 10(1):133, 2022.

E. Gordon-Rodriguez, T. Quinn, and J. P. Cunningham. Data augmentation for compositional data: Advancing predictive models of the microbiome. Advances in Neural Information Processing Systems, 35:20551–20565, 2022.

M. He, N. Zhao, and G. A. Satten. MIDASim: a fast and simple simulator for realistic microbiome data. Microbiome, 12(1):135, 2024.

Y. Jiang, M. Atton, Q. Zhu, and Y. Y. Lu. MIOSTONE: Modeling microbiome-trait associations with taxonomy-adaptive neural networks. bioRxiv, pages 2023–11, 2023.

F. Pedregosa, G. Varoquaux, A. Gramfort, V. Michel, B. Thirion, O. Grisel, M. Blondel, P. Prettenhofer, R. Weiss, V. Dubourg, J. Vanderplas, A. Passos, D. Cournapeau, M. Brucher, M. Perrot, and E. Duchesnay. Scikit-learn: Machine learning in Python. Journal of Machine Learning Research, 12:2825–2830, 2011.

R. Rong, S. Jiang, L. Xu, G. Xiao, Y. Xie, D. Liu, Q. Li, and X. Zhan. MB-GAN: microbiome simulation via generative adversarial network. GigaScience, 10(2):giab005, 2021.

E. Sayyari, B. Kawas, and S. Mirarab. TADA: phylogenetic augmentation of microbiome samples enhances phenotype classification. Bioinformatics, 35(14):i31–i40, 2019.

H. Zhang, M. Cisse, Y. N. Dauphin, and D. Lopez-Paz. mixup: Beyond empirical risk minimization. International Conference on Learning Representations, 2018.

Q. Zhu, U. Mai, W. Pfeiffer, S. Janssen, F. Asnicar, J. G. Sanders, P. Belda-Ferre, G. A. Al-Ghalith, E. Kopylova, D. McDonald, et al. Phylogenomics of 10,575 genomes reveals evolutionary proximity between domains Bacteria and Archaea. Nature Communications, 10(1):5477, 2019.
