# Supplementary material for: PhyloMix: enhancing microbiome-trait association prediction through phylogeny-mixing augmentation
Source: Bioinformatics. 2025 Jan 12;41(2):btaf014. doi: 10.1093/bioinformatics/btaf014 (PMC11849959; doi:10.1093/bioinformatics/btaf014)
Supplement: btaf014_Supplementary_Data [file btaf014_supplementary_data.zip › OP-CBIO250014.docx]

Jiang *et al.*

PhyloMix[AQ1]

Genome analysis

**PhyloMix: enhancing microbiome-trait association prediction through phylogeny-mixing augmentation**

Yifan Jiang1, Disen Liao1, Qiyun Zhu2, Yang Young Lu1,*[AQ2]

1Cheriton School of Computer Science, University of Waterloo, Waterloo, ON, Canada[AQ3]

2School of Life Sciences, Arizona State University, Tempe, AZ, United States[AQ4]

*Corresponding author. E-mail: yanglu@uwaterloo.ca[AQ5][AQ6]

Associate Editor: Russell Schwartz

**Abstract**

**Motivation:** Understanding the associations between traits and microbial composition is a fundamental objective in microbiome research. Recently, researchers have turned to machine learning (ML) models to achieve this goal with promising results. However, the effectiveness of advanced ML models is often limited by the unique characteristics of microbiome data, which are typically high-dimensional, compositional, and imbalanced. These characteristics can hinder the models’ ability to fully explore the relationships among taxa in predictive analyses. To address this challenge, data augmentation has become crucial. It involves generating synthetic samples with artificial labels based on existing data and incorporating these samples into the training set to improve ML model performance.

**Results:** Here, we propose PhyloMix, a novel data augmentation method specifically designed for microbiome data to enhance predictive analyses. PhyloMix leverages the phylogenetic relationships among microbiome taxa as an informative prior to guide the generation of synthetic microbial samples. Leveraging phylogeny, PhyloMix creates new samples by removing a subtree from one sample and combining it with the corresponding subtree from another sample. Notably, PhyloMix is designed to address the compositional nature of microbiome data, effectively handling both raw counts and relative abundances. This approach introduces sufficient diversity into the augmented samples, leading to improved predictive performance. We empirically evaluated PhyloMix on six real microbiome datasets across five commonly used ML models. PhyloMix significantly outperforms distinct baseline methods including sample-mixing-based data augmentation techniques like vanilla mixup and compositional cutmix, as well as the phylogeny-based method TADA. We also demonstrated the wide applicability of PhyloMix in both supervised learning and contrastive representation learning.

**Availability** **and implementation:** The Apache-licensed source code is available at (https://github.com/batmen-lab/phylomix).

**1 Introduction**

[AQ7]The human microbiome represents the intricate communities of microorganisms residing in and on our bodies, with bacteria alone possessing 100 times more unique genes than the human genome (Qin *et al*. [2010](file:///\\chenassoft\SmartEdit\WatchFolder\XML_Signal_to_CCE_High_Speed_WF\ESA\IN\INPROCESS\29)). These microbiomes play a crucial role in human physiology, significantly affecting both health and disease (Sekirov *et al*. [2010](file:///\\chenassoft\SmartEdit\WatchFolder\XML_Signal_to_CCE_High_Speed_WF\ESA\IN\INPROCESS\34)). For example, the microbiome influences a wide range of diseases, including diabetes (Qin *et al*. [2012](file:///\\chenassoft\SmartEdit\WatchFolder\XML_Signal_to_CCE_High_Speed_WF\ESA\IN\INPROCESS\30)), obesity (Turnbaugh *et al*. [2006](file:///\\chenassoft\SmartEdit\WatchFolder\XML_Signal_to_CCE_High_Speed_WF\ESA\IN\INPROCESS\37)), inflammatory bowel disease (Mills *et al*. [2022](file:///\\chenassoft\SmartEdit\WatchFolder\XML_Signal_to_CCE_High_Speed_WF\ESA\IN\INPROCESS\26)), and even neurodegenerative conditions such as Parkinson’s disease (Dutta *et al*. [2019](file:///\\chenassoft\SmartEdit\WatchFolder\XML_Signal_to_CCE_High_Speed_WF\ESA\IN\INPROCESS\12)) and Alzheimer’s disease (Vogt *et al*. [2017](file:///\\chenassoft\SmartEdit\WatchFolder\XML_Signal_to_CCE_High_Speed_WF\ESA\IN\INPROCESS\38)). Exploring the association between the microbiome and host traits will offer insights into the mechanisms by which the microbiome influences human health and disease, paving the way for the development of innovative therapeutic strategies.

To investigate microbiome-trait associations, researchers have employed machine learning (ML) models to uncover complex patterns within microbial samples under various conditions and thereby facilitate microbiome-based disease detection (McCoubrey *et al*. [2021](file:///\\chenassoft\SmartEdit\WatchFolder\XML_Signal_to_CCE_High_Speed_WF\ESA\IN\INPROCESS\24)). Disease prediction from microbial samples is typically framed as a supervised learning task. In this approach, an ML model is trained on microbial samples—often characterized by the abundance of selected microbial taxa—to predict a host trait of interest, which may be binary (e.g.disease status) or continuous (e.g.age). Extensive studies have shown that ML models hold significant potential for accurately classifying clinically relevant traits across various diseases using microbial sample profiles (Wu *et al*. [2021](file:///\\chenassoft\SmartEdit\WatchFolder\XML_Signal_to_CCE_High_Speed_WF\ESA\IN\INPROCESS\40), Medina *et al*. [2022](file:///\\chenassoft\SmartEdit\WatchFolder\XML_Signal_to_CCE_High_Speed_WF\ESA\IN\INPROCESS\25)). These ML models range from classic approaches like logistic regression and support vector machines to more advanced techniques like random forests and deep neural networks.

Despite increasing interest, the effectiveness of advanced ML models has been constrained by the unique characteristics of microbiome data, limiting their ability to fully explore the relationships among taxa in predictive analyses. Specifically, microbiome features, which represent the abundance of hundreds to thousands of taxa, are inherently high-dimensional. This high dimensionality presents analytical challenges, as the relatively small number of samples can result in overfitting during training, thus limiting the model’s ability to generalize to unseen samples or even leading to conflicting research conclusions (Knights *et al*. [2011](file:///\\chenassoft\SmartEdit\WatchFolder\XML_Signal_to_CCE_High_Speed_WF\ESA\IN\INPROCESS\20), Reiman *et al*. [2020](file:///\\chenassoft\SmartEdit\WatchFolder\XML_Signal_to_CCE_High_Speed_WF\ESA\IN\INPROCESS\31)). Furthermore, microbiome data is inherently compositional because the profiling instrument imposes an arbitrary total on the profiled taxa abundances (Gloor *et al*. [2017](file:///\\chenassoft\SmartEdit\WatchFolder\XML_Signal_to_CCE_High_Speed_WF\ESA\IN\INPROCESS\13)). This can lead to issues when compositional data is analyzed using noncompositional methods. Lastly, the host traits of interest are often unevenly distributed, with training data either over-representing diseased states and lacking sufficient healthy samples, or vice versa, resulting in a distribution that does not accurately reflect the general population. Thus, new methods are required to adapt machine learning models to complex data challenges, including low sample sizes, high feature dimensionality, compositionality, and unbalanced trait distribution.

Ideally, ML models could be better trained for predictive analyses with more microbial samples, but in reality, collecting these samples is laborious and costly. From a practitioner’s perspective, a more cost-effective alternative is to computationally augment the data, an approach that has been increasingly studied by the ML community in recent years (Mumuni and Mumuni [2022](file:///\\chenassoft\SmartEdit\WatchFolder\XML_Signal_to_CCE_High_Speed_WF\ESA\IN\INPROCESS\27)). The core concept of data augmentation is to generate synthetic samples with artificial labels based on existing data, and incorporate these into the training data to enhance ML model performance [AQ8](Fig. 1a). For example, one of the pioneering methods, SMOTE (Chawla *et al*. [2002](file:///\\chenassoft\SmartEdit\WatchFolder\XML_Signal_to_CCE_High_Speed_WF\ESA\IN\INPROCESS\8)), generates synthetic samples by oversampling minority classes using k-nearest intra-class neighbors. More recently, mixup (Zhang *et al*. [2018](file:///\\chenassoft\SmartEdit\WatchFolder\XML_Signal_to_CCE_High_Speed_WF\ESA\IN\INPROCESS\43)) and its variants (Cao *et al*. 2022) create synthetic samples by linearly combining two samples and their corresponding labels, which are drawn randomly from the training data. However, most previous works are general-purpose and not tailored for microbiome data, overlooking domain-specific knowledge unique to the microbiome. Numerous approaches have been developed to augment microbiome data, with generative methods such as PhylaGAN (Sharma *et al*. [2024](file:///\\chenassoft\SmartEdit\WatchFolder\XML_Signal_to_CCE_High_Speed_WF\ESA\IN\INPROCESS\36)), MB-GAN (Rong *et al*. [2021](file:///\\chenassoft\SmartEdit\WatchFolder\XML_Signal_to_CCE_High_Speed_WF\ESA\IN\INPROCESS\32)), and TADA (Sayyari *et al*. [2019](file:///\\chenassoft\SmartEdit\WatchFolder\XML_Signal_to_CCE_High_Speed_WF\ESA\IN\INPROCESS\33)) standing out as notable advancements. These techniques aim to capture the data’s underlying distributional characteristics. While GAN-based methods are powerful, they often face challenges in effectively training on small-scale datasets due to their high sensitivity to data quantity and quality. To our knowledge, the only exception is TADA (Sayyari *et al*. [2019](file:///\\chenassoft\SmartEdit\WatchFolder\XML_Signal_to_CCE_High_Speed_WF\ESA\IN\INPROCESS\33)), but this algorithm is limited to mimicking sample-wise variations and fails to fully explore the potential of advanced data augmentation techniques (see detailed comparison in Section 4).[AQ9]

In this study, we introduce a novel data augmentation method specifically designed for microbiome data to enhance predictive analyses, called Phylogeny-Mixing augmentation (PhyloMix). The key ideas behind PhyloMix is to leverage the phylogenetic relationships among microbiome taxa, using them as an informative prior to guide the generation of synthetic microbial samples. Here, phylogeny serves as a tool to organize and understand microbial taxa by encoding their evolutionary relationships. Leveraging phylogeny, PhyloMix generates new samples by removing a subtree from one sample and recombining the counterpart from another sample, rather than interpolating between two whole microbial samples. This approach considers that variations among microbiome taxa are constrained by their evolutionary relationships as encoded by the phylogeny, thereby producing more realistic synthetic samples. Importantly, PhyloMix encodes phylogenetic relationships using a novel phylogenetic probabilistic profiling to address the compositional nature of microbiome data, allowing it to effectively handle both raw counts and relative abundances.

We have evaluated PhyloMix for supervised learning tasks using three simulated and six publicly available microbiome datasets, each with varying sample sizes and feature dimensionality. We also benchmarked the evaluation using five commonly used ML models, ranging from classic logistic regression and support vector machines to more advanced methods like random forests and deep neural networks. Overall, the augmentation generated by PhyloMix preserves or notably enhances predictive performance, regardless of the dataset complexity or the sophistication of the ML models used. In addition to supervised learning, PhyloMix enables representation learning by using randomly augmented training samples to define a self-supervised optimization objective, pulling random augmentations of the same sample closer together while pushing those generated from different samples further apart (Chen *et al*. [2020](file:///\\chenassoft\SmartEdit\WatchFolder\XML_Signal_to_CCE_High_Speed_WF\ESA\IN\INPROCESS\9)). We observe that PhyloMix outperforms previous representation learning approaches by identifying more distinguishable representations of samples with different host trait labels.

**2 Approach**

**2.1 Problem setup**

Consider a supervised learning task involving a training data of *n* microbial samples, represented as a data matrix. The rows of the data matrix correspond to the set of samples, denoted as where represents the *P*-dimensional features of the *i*th sample. The columns of the data matrix represent the set of features corresponding to the abundance of microbial taxa, denoted as where represents the abundance of the *j*th taxon observed across all samples. The abundance of microbial taxa can be measured either by count or by normalized probability. The training data also includes trait labels, denoted as . Each sample is paired with a corresponding trait label , which is binary(e.g.disease status) or continuous (e.g.age).

In addition, we have phylogenetic relationships among microbial taxa that encode their evolutionary dependencies. The phylogeny classifies taxa through a series of splits, representing estimated events where two lineages diverge from a common ancestor to form distinct species (Washburne *et al*. [2018](file:///\\chenassoft\SmartEdit\WatchFolder\XML_Signal_to_CCE_High_Speed_WF\ESA\IN\INPROCESS\39)). Two taxa with close phylogenetic relationships are likely to have similar functional roles, exhibiting more similar abundance variations compared to two randomly selected taxa. PhyloMix uses an inferred binary phylogenetic tree (see Section 3.2), denoted as , with the tree’s leaf nodes labeled by the set of taxa . The tree’s internal nodes are indexed from 1 to in a top-down fashion, denoted as .

Given the samples , the corresponding trait labels , as well as the phylogenetic tree , our goal is to generate synthetic samples with corresponding synthetic trait labels , where and is the augmentation ratio specified by the user. Empowered by the augmented samples with labels , a more effective ML model should be learned (Fig. 1a), mapping a sample to the trait label , which may no longer remains binary after the data augmentation.

**2.2 Sample mixup**

PhyloMix draws inspiration from mixup (Zhang *et al*. [2018](file:///\\chenassoft\SmartEdit\WatchFolder\XML_Signal_to_CCE_High_Speed_WF\ESA\IN\INPROCESS\43)), a widely used data augmentation technique that creates synthetic samples by linearly combining two randomly drawn samples from the same dataset and their corresponding labels. Specifically, given two random drawn sample-label pairs and , where denotes the input samples and are their corresponding trait labels, mixup a new synthetic sample by:

(1)

where is the mixing weight drawn from a beta distribution. It is worth noting that mixup is designed to be generic, treating the features corresponding to the abundance of microbial taxa equally. Considering that recent studies have leveraged the inherent correlation structure among taxa as an informative prior to improve disease prediction performance (Albanese *et al*. [2015](file:///\\chenassoft\SmartEdit\WatchFolder\XML_Signal_to_CCE_High_Speed_WF\ESA\IN\INPROCESS\3), Xiao *et al*. [2018](file:///\\chenassoft\SmartEdit\WatchFolder\XML_Signal_to_CCE_High_Speed_WF\ESA\IN\INPROCESS\41), Reiman *et al*. [2020](file:///\\chenassoft\SmartEdit\WatchFolder\XML_Signal_to_CCE_High_Speed_WF\ESA\IN\INPROCESS\31), Sharma *et al*. [2020](file:///\\chenassoft\SmartEdit\WatchFolder\XML_Signal_to_CCE_High_Speed_WF\ESA\IN\INPROCESS\35), Jiang *et al*. 2023), we hypothesize that a data augmentation technique specifically tailored for microbiome data by incorporating this information can further enhance predictive accuracy.

**2.3 Constructing sample-specific phylogenetic profiles**

PhyloMix aims to enhance mixup-based data augmentation by incorporating phylogenetic relationships among microbiome taxa to guide the generation of synthetic samples. We are aware of the mixup variation that uses constituency parsing trees to generate synthetic sentences in the natural language processing community (Zhang *et al*. [2022](file:///\\chenassoft\SmartEdit\WatchFolder\XML_Signal_to_CCE_High_Speed_WF\ESA\IN\INPROCESS\44)). However, this method cannot be directly applied to microbiome data due to its unique characteristics. Specifically, each microbial sample is represented by a set of features corresponding to the abundance of microbial taxa, measured either by count or normalized probability. In the case of count data, two samples may be profiled under different sequencing depths. Thus, a higher count measured for taxon A in sample 1 compared to taxon B in sample 2 may result either from taxon A being more highly expressed than B or from sample 1 having a much higher overall sequencing depth than sample 2. In the case of normalized probability, where counts across taxa are scaled to sum to 1, substituting the probability of taxon A in sample 1 with that of sample 2 may violate the sum-to-one constraint in sample 1, rendering the resultant features of the synthetic sample invalid.

To address this challenge, we construct a probabilistic phylogenetic profile for each sample, ensuring that it remains valid to remove features corresponding to an arbitrary subtree from one sample and recombine them with the counterpart from another sample (Fig. 1b). Specifically, the probabilistic phylogenetic profile of a sample *x* includes the following information: (i) the total abundance , where indicates the abundance of the *j*th taxon in sample *x*, and (ii) the phylogenetic probability for each internal node in the phylogenetic tree, where and indicate the relative abundance of the descendants of its left and right children, respectively, with :

(2)

where and denote the left and right children of *v*, and denotes the descendent leaf nodes of *v*.

With the probabilistic phylogenetic profile of *x*, we can reconstruct the relative and absolute abundance for each microbiome taxon by traversing the phylogenetic tree in a top-down fashion. Specifically, for the *j*th taxon, we trace the path from the root to the leaf node and calculate its relative abundance by multiplying the phylogenetic probabilities of the internal nodes encountered along the path. For example, as illustrated in Fig. 1b, the relative abundance of the first taxon in sample 1 is calculated as . Similarly, the relative abundance of the last taxon in sample 1 is calculated as . Given the total abundance of *x* across all taxa, the absolute abundance for the *j*th taxon is simply the allocation of this total abundance to that taxon. For example, the absolute abundance of the first and last taxa in sample 1 is calculated as and , respectively.

**2.4 Generating synthetic samples**

With probabilistic phylogenetic profiles enabling valid exchange of arbitrary subtree between samples, we can now leverage phylogenetic information to guide the generation of synthetic microbial samples. Concretely, for a given sample-label pair , we randomly sample another sample-label pair from the training data. First, we construct probabilistic phylogenetic profiles for both samples, allowing us to sample subtrees for exchange. Next, we draw a mixing weight from a beta distribution , where determines whether the synthetic sample combines evenly from the two original samples or is biased toward one of them. Intuitively, we want the portion of taxa exchanged to be reasonable. If the portion exchanged is too small or too large, the exchange may closely resemble the original samples and fail to introduce sufficient diversity into the augmented sample. Therefore, we use a distribution in this study, ensuring that the mixing weights drawn are centered around 0.5 (see Section 4.3 for the robustness of different Beta distribution options).

To ensure that the taxa exchanged are constrained by the phylogenetic tree, we employ a two-phase sampling strategy. Specifically, We randomly draw an internal node from the phylogenetic tree and exchange the phylogenetic profiles between and for all descendent internal nodes of *v*, provided they have not already been exchanged. We repeat this draw-and-exchange process the number of exchanged internal nodes approximately reaches . Once we have the mixed probabilistic phylogenetic profiles, with internal nodes originally belonging to sample and to sample , we can reconstruct the relative and absolute abundance for each microbiome taxon based on the total abundance , as described in Section 2.3. We denote the synthetic sample with the generated absolute abundance values as , and create its label as . We repeat the process of generating synthetic samples until we have new samples, where is the augmentation ratio specified by the user. We use in this study (see Section 4.3 for the robustness of different options).

**3 Materials and methods**

**3.1 Datasets**

We conducted a comprehensive evaluation of PhyloMix using six publicly available microbiome datasets with varying sample sizes and feature dimensionality (details in Supplementary Table S1). The AlzBiom dataset (Laske *et al*. [2022](file:///\\chenassoft\SmartEdit\WatchFolder\XML_Signal_to_CCE_High_Speed_WF\ESA\IN\INPROCESS\21)) investigated the connection between the gut microbiome and Alzheimer’s disease (AD). It includes 75 amyloid-positive AD samples and 100 cognitively healthy control samples from the AlzBiom study, with profiles containing *P* = 8350 taxa. The ASD dataset (Dan *et al*. [2020](file:///\\chenassoft\SmartEdit\WatchFolder\XML_Signal_to_CCE_High_Speed_WF\ESA\IN\INPROCESS\10)) examined the relationship between the gut microbiome and abnormal metabolic activity in individuals with Autism Spectrum Disorder (ASD). It consists of 30 typically developing (TD) and 30 constipated ASD (C-ASD) samples, with profiles containing *P* = 7287 taxa. The GD dataset (Zhu *et al*. [2021](file:///\\chenassoft\SmartEdit\WatchFolder\XML_Signal_to_CCE_High_Speed_WF\ESA\IN\INPROCESS\46)) investigated the link between the gut microbiome and Graves’ disease (GD). It includes 100 GD samples and 62 healthy control samples, with profiles containing *P* = 8487 taxa. The RUMC dataset (Boktor *et al*. [2023](file:///\\chenassoft\SmartEdit\WatchFolder\XML_Signal_to_CCE_High_Speed_WF\ESA\IN\INPROCESS\5)) is part of a cohort study exploring the relationship between the gut microbiome and the Parkinson’s disease (PD). The dataset includes 42 PD samples and 72 healthy control samples, with profiles containing *P* = 7256 taxa. The IBD dataset (Gonzalez *et al*. [2022](file:///\\chenassoft\SmartEdit\WatchFolder\XML_Signal_to_CCE_High_Speed_WF\ESA\IN\INPROCESS\15)) studied the relationship between the gut microbiome and two main subtypes of inflammatory bowel disease (IBD): Crohn’s disease (CD) and ulcerative colitis (UC). It includes 108 CD and 66 UC samples, with profiles containing *P* = 5287 taxa. The HMP2 dataset (Lloyd-Price *et al*. [2019](file:///\\chenassoft\SmartEdit\WatchFolder\XML_Signal_to_CCE_High_Speed_WF\ESA\IN\INPROCESS\22)), part of the Integrative Human Microbiome Project (iHMP) [Research Network Consortium (2014), iHMP], also explored the relationship between the gut microbiome and two IBD subtypes: CD and UC. Compared to the IBD dataset, this dataset increases the sample size to *n* = 1158 (728 CD and 430 UC samples) and expands the taxa set to *P* = 10 614.

In addition, we created three simulated datasets using the microbiome data simulator MIDASim (He *et al*. [2024](file:///\\chenassoft\SmartEdit\WatchFolder\XML_Signal_to_CCE_High_Speed_WF\ESA\IN\INPROCESS\17)). We used a microbiome data simulator MIDASim (He *et al*. [2024](file:///\\chenassoft\SmartEdit\WatchFolder\XML_Signal_to_CCE_High_Speed_WF\ESA\IN\INPROCESS\17)) to generate simulated data by leveraging a template microbiome dataset while preserving its correlation structure to maintain similarity. We selected the IBD dataset as the template and adjusted the simulation settings to create three datasets with varying levels of difficulty in distinguishing between labels. Since MIDASim is not designed to generate simulated samples with labels, we selected a real dataset with positive and negative labels. We then simulated samples separately for each label (positive and negative) before combining them into a unified simulated dataset (refer to Supplementary Section S2 for the details of simulated dataset generation).

**3.2 Phylogenetic tree**

The abundance features in these datasets were profiled using Metagenomic Shotgun (MGS) sequencing, adhering to the standard operating procedures for shotgun metagenomics as implemented on the Qiita platform (Gonzalez *et al*. [2018](file:///\\chenassoft\SmartEdit\WatchFolder\XML_Signal_to_CCE_High_Speed_WF\ESA\IN\INPROCESS\14)). Specifically, the raw sequencing reads were classified using Woltka v0.1.4 (Zhu *et al*. [2022](file:///\\chenassoft\SmartEdit\WatchFolder\XML_Signal_to_CCE_High_Speed_WF\ESA\IN\INPROCESS\47)) against the Web of Life (WoL) v2 database (Zhu *et al*. [2019](file:///\\chenassoft\SmartEdit\WatchFolder\XML_Signal_to_CCE_High_Speed_WF\ESA\IN\INPROCESS\45)), which includes 15 953 microbial genomes. Note that a small number of internal nodes in the WoL phylogeny have more than two lineages (i.e.polytomies). To address this, we resolved the issue by creating an arbitrary dichotomous structure among the affected nodes (refer to Supplementary Section S4 for the robustness of different polytomy resolution strategies). Furthermore, because each dataset only profiles a subset of taxa in the WoL phylogeny, we used a pruned phytogenetic tree having the dataset-specific taxa as leaf nodes only.

**3.3 Evaluation tasks**

We assessed the effectiveness of PhyloMix in two distinct settings: supervised learning and representation learning. The supervised learning setting evaluates PhyloMix’s ability to generate augmented samples that improve predictive performance for trait labels. To assess the predictive performance, we employed two metrics: the Area Under the Receiver Operating Characteristic Curve (AUROC) and the Area Under the Precision-Recall Curve (AUPRC). Both metrics are widely used to evaluate predictive performance, with AUPRC being particularly well-suited for imbalanced datasets (Davis and Goadrich [2006](file:///\\chenassoft\SmartEdit\WatchFolder\XML_Signal_to_CCE_High_Speed_WF\ESA\IN\INPROCESS\11)).

The representation learning setting assesses PhyloMix’s capability to generate augmented samples that facilitate the extraction of meaningful high-level features from raw data (Bengio *et al*. [2013](file:///\\chenassoft\SmartEdit\WatchFolder\XML_Signal_to_CCE_High_Speed_WF\ESA\IN\INPROCESS\4)). PhyloMix adopts a contrastive learning-based representation learning approach. Specifically, PhyloMix uses the same encoder architecture as the deep autoencoder, mapping representations to a space where the contrastive loss is applied (Chen *et al*. [2020](file:///\\chenassoft\SmartEdit\WatchFolder\XML_Signal_to_CCE_High_Speed_WF\ESA\IN\INPROCESS\9)). The contrastive loss function is designed to bring the representations of positive pairs of samples closer together while pushing the representations of negative pairs further apart. Here, positive pairs are defined as two synthetic samples generated by applying random data augmentations to the same training sample pairs, whereas negative pairs are synthetic samples generated from different sample pairs. To evaluate representation learning performance, we utilized the learned representations as input for a downstream classifier to carry out predictive tasks.

**3.4 Benchmark settings**

We incorporated PhyloMix with five ML models with varying predictive capabilities: logistic regression (LR), support vector machine (SVM) with a linear kernel, random forest (RF), multi-layer perceptron (MLP), and MIOSTONE, a state-of-the-art deep learning model that encodes taxonomy (Jiang *et al*. 2023). We used the Scikit-learn implementation (Pedregosa *et al*. [2011](file:///\\chenassoft\SmartEdit\WatchFolder\XML_Signal_to_CCE_High_Speed_WF\ESA\IN\INPROCESS\28)) with default settings for the LR, RF, and SVM models. The MLP model was configured with a pyramid-shaped architecture featuring two hidden layers of size 256 and 128, respectively. For the MIOSTONE model, we employed the default implementation settings (refer to Supplementary Section S3 for the details of ML model settings and trainings). For all methods, we preprocessed microbiome features using centered log-ratio transformation (Aitchison [1982](file:///\\chenassoft\SmartEdit\WatchFolder\XML_Signal_to_CCE_High_Speed_WF\ESA\IN\INPROCESS\2)) prior to data augmentation (refer to Supplementary Sections S3 and S6.2 for the robustness of using other types of data transformation such as relative abundance normalization).

It is important to note that while the trait labels for the training samples are binary, they become continuous for the generated synthetic samples. For deep learning models like MLP and MIOSTONE, training is based on cross-entropy loss, so the continuous labels will not affect the learning process. Continuous labels provide more robust predictions, similar to the role of label smoothing (Zhang *et al*. [2021](file:///\\chenassoft\SmartEdit\WatchFolder\XML_Signal_to_CCE_High_Speed_WF\ESA\IN\INPROCESS\42)). For other models like LR, SVM, and RF, which are designed for discrete labels, we instead use their regression counterparts–linear regression, support vector regression, and random forest regression–to train on the augmented data with continuous labels. We also utilized the Scikit-learn implementation (Pedregosa *et al*. [2011](file:///\\chenassoft\SmartEdit\WatchFolder\XML_Signal_to_CCE_High_Speed_WF\ESA\IN\INPROCESS\28)) with default settings for these regression models.

**3.5 Baseline methods**

We compared PhyloMix’s performance against several distinct baseline methods, each generating an equivalent amount of synthetic samples to train the ML models alongside the original training data. The vanilla mixup (Zhang *et al*. [2018](file:///\\chenassoft\SmartEdit\WatchFolder\XML_Signal_to_CCE_High_Speed_WF\ESA\IN\INPROCESS\43)), a classic data augmentation method, creates synthetic samples by linearly combining two samples along with their corresponding labels. The compositional cutmix (Gordon-Rodriguez *et al*. [2022](file:///\\chenassoft\SmartEdit\WatchFolder\XML_Signal_to_CCE_High_Speed_WF\ESA\IN\INPROCESS\16)) extends the vanilla mixup to accommodate compositional data. TADA (Sayyari *et al*. [2019](file:///\\chenassoft\SmartEdit\WatchFolder\XML_Signal_to_CCE_High_Speed_WF\ESA\IN\INPROCESS\33)) generates synthetic samples by explicitly modeling biological and sampling variations, guided by the evolutionary relationships among taxa. MB-GAN (Rong *et al*. [2021](file:///\\chenassoft\SmartEdit\WatchFolder\XML_Signal_to_CCE_High_Speed_WF\ESA\IN\INPROCESS\32)) employs generative adversarial networks to learn from microbial abundance data and generate simulated abundances that closely mimic the original data.

**4 Results**

**4.1 PhyloMix enhances supervised learning**

We evaluated data augmentation performance on three simulated and six real microbiome datasets with varying sample and microbial taxa sizes. These datasets cover a range of disease prediction tasks, including Autism Spectrum Disorder, Alzheimer’s disease, Graves’ disease, Parkinson’s disease, and inflammatory bowel disease. Thus, they provide a comprehensive evaluation for predictive models of the human gut microbiome.

We evaluated the predictive performance using 5-fold cross-validation, training individual models on each training split and testing them on the corresponding test splits. The predictions from all test splits were concatenated, and the AUROC and AUPRC scores were calculated on the full dataset. For robustness, we repeated this process 20 times using different random seeds and reported the mean performance along with the 95% confidence intervals. Each repetition involved both data splitting and ML model training.

Our analysis shows that the augmentation generated by PhyloMix preserves or notably enhances predictive performance (Fig. 2a and Supplementary Section S6.1), regardless of the dataset complexity or the sophistication of the ML models used. On average across all ML models, PhyloMix achieves a , , and improvement in test AUPRC on three simulated datasets. In comparison, vanilla mixup, the second-best data augmentation method, yields only a , , and improvement in test AUPRC on three simulated datasets. For real datasets, PhyloMix achieves a , , , and improvement in test AUPRC on the RUMC, AlzBiom, IBD, and ASD datasets, respectively. In comparison, vanilla mixup yields only a , , , and improvement in test AUPRC on the RUMC, AlzBiom, IBD, and ASD datasets, respectively. Importantly, PhyloMix rarely hurt model performance, with drops observed in only 2 out of 35 cases, and the average decrease is typically just . In comparison, vanilla mixup shows decreased performance in 8 out of 35 cases, with an average reduction of . In the remaining GD and HMP2 datasets, PhyloMix achieves only a modest improvement of and in test AUPRC, as these datasets already exhibit near-perfect predictive performance, regardless of data augmentation. To rigorously quantify how much better is PhyloMix compared to the vanilla mixup, we compared the performance between PhyloMix and the vanilla mixup using one-tailed two-sample t-tests to calculate *P*-values. PhyloMix demonstrates significantly better performance (*P*-value ≤ 0.05) in terms of test AUPRC on five out of eight datasets with the logistic regression model, seven out of eight datasets with the support vector machine model, four out of eight datasets with the random forest model, two out of eight datasets with the multi-layer perceptron model, and four out of eight datasets with the MIOSTONE model. In other words, PhyloMix performs at least as well as vanilla mixup and is significantly better in more than half of the scenarios. A similar conclusion can be drawn based on AUROC performance (Supplementary Figs S4 and S6).

Finally, in addition to the quantitative comparison, we conducted a qualitative evaluation of PhyloMix and other baseline methods (Fig. 2b). We directly visualize the improvement in predictive performance after applying each data augmentation method to generate synthetic training samples. The results demonstrate that PhyloMix consistently ranks among the top performers in both metrics across all datasets and various ML models. This is evident from the radar plots (Fig. 2b), where PhyloMix’s contours are always the largest and encompass those of other methods as subsets. We observed that TADA, which relies on modeling biological and sampling variations, fails to noticeably improve model performance, despite using the same phylogenetic information as PhyloMix. This underscores the importance of employing sample and label mixing for effective synthetic sample generation. Compositional cutmix and vanilla mixup can fail to enhance model performance when applied to LR and MIOSTONE models, highlighting their limitations in applicability. Importantly, PhyloMix consistently improves the performance of all ML models across different random seeds, regardless of the model’s complexity.

**4.2 PhyloMix enhances representation learning**

Having demonstrated improvements in predicting trait labels, we next evaluated PhyloMix’s capability to generate augmented samples that facilitate the extraction of meaningful high-level features from raw data. We compared PhyloMix’s performance against three alternative data augmentation methods: the vanilla mixup, the compositional cutmix, and TADA. In addition, since the UniFrac distance is a widely used metric for comparing microbial samples (Lozupone and Knight [2005](file:///\\chenassoft\SmartEdit\WatchFolder\XML_Signal_to_CCE_High_Speed_WF\ESA\IN\INPROCESS\23)), we included an additional contrastive learning-based baseline, where positive and negative sample pairs were selected based on their UniFrac distance, with positive pairs having a smaller UniFrac distance compared to negative pairs. Furthermore, traditional representation learning typically employs a deep autoencoder to derive low-dimensional representations by minimizing reconstruction loss. For comparison, we included a deep autoencoder as a baseline method, where the encoder consists of three hidden layers with sizes of 512, 256, and 128, respectively. The decoder mirrors this architecture in reverse.

We assessed the quality of the learned representations using the following evaluation criteria. The learned representation from all methods is input into a logistic regression model to predict the trait label, using the same procedure as in the supervised learning setting. For robustness, we repeated this process 20 times using different random seeds and reported the mean performance along with the 95% confidence intervals. Each repetition involved data splitting, representation learning, and ML model training.

Our analysis reveals that PhyloMix’s contrastive model consistently outperforms both the deep autoencoder and the contrastive models that use alternative data augmentation methods in terms of learned representation quality (Fig. 3). On average, PhyloMix achieves a and improvement in test AUPRC and AUROC compared to using the representation learned by deep autoencoder, respectively. Importantly, PhyloMix enhances the quality of learned representations across all datasets, with the smallest improvement observed being in test AUPRC and in AUROC on the RUMC dataset. In comparison, compositional cutmix, the second-best data augmentation method in representation learning, shows only an improvement in test AUPRC and an improvement in AUROC over the deep autoencoder. Although UniFrac distance is widely used for comparing microbial samples based on phylogeny, PhyloMix proves to be more effective in leveraging phylogeny as an informative prior to enhance the quality of representation learning. The results demonstrate that PhyloMix consistently ranks among the top performers in both metrics across all datasets and various ML models.

**4.3 Dissecting the performance of PhyloMix**

We first evaluated the computational cost of using PhyloMix in conjunction with five ML models with different levels of complexities. For a fair comparison, all models were trained and tested in the same environment: AMD EPYC 7302 16-Core Processor, NVIDIA RTX A6000 GPU, with 32GB DDR4 RAM. PhyloMix’s data augmentation involves two primary components: generating synthetic samples and the additional training time necessary to integrate these synthetic samples. We found that PhyloMix’s sample generation incurs only moderate computational overhead, measured in seconds (Fig. 4a and Supplementary Fig. S10). The additional training time required to incorporate these synthetic samples is marginal on the AlzBiom dataset, except for the HMP2 dataset, which has a larger sample size of *n* = 1158 and higher dimensionality with *P* = 10 614 taxa. Note that when the sample size is large, data augmentation becomes less critical, as the datas*et al*ready exhibits near-perfect predictive performance, with or without augmentation. However, when the sample size is small, the computational time is negligible compared to the improvement in predictive accuracy gained through augmentation.

PhyloMix’s data augmentation relies on two factors: the choice of the sample-mixing distribution and the augmentation level. To evaluate the impact of each component on predictive performance, we conducted a control study using the AlzBiom dataset by modifying PhyloMix, altering its components with different variations Specifically, we considered two variants of PhyloMix: (i) altering the Beta distributions that control sample mixing weights; (ii) adjusting the number of augmentation samples added. The results indicate that PhyloMix is robust to the choice of these two factors. In the first study (Fig. 4b), we compared three Beta distribution options, each representing a distinct sample mixing strategy. Specifically, tends to bias the synthetic sample toward one of the original samples, encourages a more even combination from both original samples, while results in a more arbitrary combination. In general, we observed that the Beta distribution choice does not negatively impact data augmentation performance. The only exception is the logistic regression model, where data augmentation transforms logistic regression into linear regression, leading to a performance downgrade if the Beta distribution is not chosen properly so that the synthetic samples may closely resemble the original ones and fail to introduce sufficient diversity.

In the second study (Fig. 4c), we compared four different augmentation levels, ranging from adding the same number of synthetic samples as the original samples to adding up to ten times as many synthetic samples. In general, we observed that data augmentation consistently improves predictive performance. To achieve a significant performance boost, we expect the number of synthetic samples added to be at least three times the number of original samples. It’s important not to include excessive synthetic samples. On one hand, performance may diminish or even drop if too many synthetic samples are added; on the other hand, increasing the number of augmented samples leads to higher computational overhead. From a theoretical perspective, mixup-based data augmentation methods such as PhyloMix are theoretically analogous to certain regularization techniques, such as label smoothing and dropout (Carratino *et al*. [2022](file:///\\chenassoft\SmartEdit\WatchFolder\XML_Signal_to_CCE_High_Speed_WF\ESA\IN\INPROCESS\7)) (refer to Supplementary Section S1 for more insights of data augmentation). From a regularization perspective, excessive regularization, such as generating an excessive number of synthetic samples, can have a detrimental effect by over-smoothing the labels, ultimately compromising prediction performance.

Furthermore, we investigate the impact of varying sample sizes on PhyloMix’s predictive performance. To address this question, we randomly selected subsets of training samples from the HMP2 dataset, with subset sizes ranging from , , , and of the total training samples, to train the ML models. For robustness, we repeated this process 20 times with different random seeds. For each taxa subset, we trained and evaluated PhyloMix using the same settings as for the full dataset. We found that while training PhyloMix with more samples generally leads to better predictive performance, the improvement provided by PhyloMix’s data augmentation is notably more significant across all ML models (Fig. 4d). It is important to note that data augmentation becomes more critical for performance when the sample size is small, highlighting the necessity of PhyloMix.

Finally, we examine the rationale behind PhyloMix’s data augmentation by visualizing its effect on smoothing the decision boundary of the SVM model. Specifically, we trained an SVM model on the training split of the IBD dataset, calculated the decision boundary, and examined how this boundary separates positive and negative samples in the test split (Fig. 4e and Supplementary Fig. S1). After data augmentation, the decision boundary shifts toward the mean of the output space, leading to a smoother and more generalized boundary. Note that the data augmentation is particularly effective at smoothing the decision boundary when the sample size is small. It is important to highlight that, beyond the empirical results, mixup-based data augmentation methods are grounded in strong theoretical principles (Carratino *et al*. [2022](file:///\\chenassoft\SmartEdit\WatchFolder\XML_Signal_to_CCE_High_Speed_WF\ESA\IN\INPROCESS\7)) (refer to Supplementary Section S1 for more insights of data augmentation).

**5 Discussion and conclusion**

In this study, we propose PhyloMix, a novel data augmentation method specifically designed for microbiome data to enhance predictive analyses. At its core, PhyloMix leverages the phylogenetic relationships among microbiome taxa as an informative prior to guide the generation of synthetic microbial samples. Leveraging phylogeny, PhyloMix generates new samples by removing a subtree from one sample and recombining the counterpart from another sample, rather than interpolating between two whole microbial samples. The key novelties of PhyloMix are 3-fold: (i) we enable valid phylogeny exchange through the use of sample-specific probabilistic phylogenetic profiles, effectively handling both raw counts and relative abundances. (ii) We demonstrate that phylogeny-based sample mixing achieves superior performance compared to relying solely on phylogeny or using sample mixing alone. (iii) We showcase the wide applicability of PhyloMix in both supervised learning and contrastive representation learning.

We evaluated PhyloMix’s performance on simulated and real microbiome datasets, each with varying sample sizes and feature dimensionality. We benchmarked the evaluation using five commonly used ML models, ranging from classic logistic regression and support vector machines to more advanced methods like random forests and deep neural networks. Overall, the augmentation generated by PhyloMix preserves or notably enhances predictive performance, regardless of the dataset complexity or the sophistication of the ML models used. Furthermore, PhyloMix significantly outperforms distinct baseline methods in supervised learning and contrastive representation learning settings, including sample-mixing-based data augmentation techniques like vanilla mixup and compositional cutmix, as well as the phylogeny-based method TADA. Importantly, our comprehensive control studies demonstrate that PhyloMix is robust with respect to the choice of sample-mixing distribution and the level of augmentation.

Lastly, this study highlights several promising directions for future research. First, we observe that generating an excessive number of synthetic samples may have a detrimental effect and ultimately compromise prediction performance. An interesting future direction would be to determine the optimal number of synthetic samples for achieving the best prediction performance in a data-driven manner. In addition, we emphasize how the synthetic data can quantitatively improve predictive performance. A potential future research direction is to explore how these synthetic data can contribute to generating meaningful biological insights and uncovering interesting biological phenomena. Finally, it is commonly believed that a better machine learning model is typically more interpretable (Adebayo *et al*. [2018](file:///\\chenassoft\SmartEdit\WatchFolder\XML_Signal_to_CCE_High_Speed_WF\ESA\IN\INPROCESS\1)). It is also observed that PhyloMix, along with other mixup-based data augmentation methods, can empirically improve the predictive performance of ML models. A promising direction for future research is to quantitatively assess the impact of data augmentation on the interpretability of machine learning models.

**Supplementary data**

Supplementary data are available at *Bioinformatics* online.[AQ10]

**Conflict of interest**

None declared.[AQ11]

**Funding**

The work was supported by the Canadian NSERC Discovery Grant [RGPIN-03270-2023].[AQ12][AQ13]

**References**

Adebayo J, Gilmer J, Muelly M *et al.* Sanity checks for saliency maps. *Adv Neural Inf Process Syst* 2018;**31**.[AQ14]

Aitchison J. The statistical analysis of compositional data. *J R Stat Soc Ser B (Methodol)* 1982;**44**:139–60. [CrossRef][[10.1111/j.2517-6161.1982.tb01195.x](http://dx.doi.org/10.1111/j.2517-6161.1982.tb01195.x)][Mismatch]

Albanese D, Filippo CD, Cavalieri D *et al.* Explaining diversity in metagenomic datasets by phylogenetic-based feature weighting. *PLoS Comput Biol* 2015;**11**:e1004186. [PMC][[10.1371/journal.pcbi.1004186](http://dx.doi.org/10.1371/journal.pcbi.1004186)] [[25815895](http://www.ncbi.nlm.nih.gov/pubmed/25815895)]

Bengio Y, Courville A, Vincent P. Representation learning: a review and new perspectives. *IEEE Trans Pattern Anal Mach Intell* 2013;**35**:1798–828. [PMC][[10.1109/TPAMI.2013.50](http://dx.doi.org/10.1109/TPAMI.2013.50)] [[23787338](http://www.ncbi.nlm.nih.gov/pubmed/23787338)]

Boktor JC, Sharon G, Verhagen Metman LA *et al.* Integrated multi-cohort analysis of the Parkinson’s disease gut metagenome. *Mov Disord* 2023;**38**:399–409. [PMC][[10.1002/mds.29300](http://dx.doi.org/10.1002/mds.29300)] [[36691982](http://www.ncbi.nlm.nih.gov/pubmed/36691982)][Mismatch] [InsertedFromOnline]

Cao C, Zhou F, Dai Y *et al.* A survey of mix-based data augmentation: taxonomy, methods, applications, and explainability. arXiv, arXiv:2212.10888, 2020, preprint: not peer reviewed.[AQ15]

Carratino L, Cissé M, Jenatton R *et al.* On mixup regularization. *J Mach Learn Res* 2022;**23**:1–31.

Chawla NV, Bowyer KW, Hall LO *et al.* SMOTE: synthetic minority over-sampling technique. *JAIR* 2002;**16**:321–57. [CrossRef][[10.1613/jair.953](http://dx.doi.org/10.1613/jair.953)]

Chen T, Kornblith S, Norouzi M *et al.* A simple framework for contrastive learning of visual representations. 2020;1597–1607.[AQ16]

Dan Z, Mao X, Liu Q *et al.* Altered gut microbial profile is associated with abnormal metabolism activity of autism spectrum disorder. *Gut Microbes* 2020;**11**:1246–67. [PMC][[10.1080/19490976.2020.1747329](http://dx.doi.org/10.1080/19490976.2020.1747329)] [[32312186](http://www.ncbi.nlm.nih.gov/pubmed/32312186)] [InsertedFromOnline]

Davis J, Goadrich M. The relationship between precision–recall and ROC curves. In: *International Conference on Machine Learning*, 2006, 233–40.[AQ17] [CrossRef][[10.1145/1143844.1143874](http://dx.doi.org/10.1145/1143844.1143874)][Mismatch]

Dutta SK, Verma S, Jain V *et al.* Parkinson’s disease: the emerging role of gut dysbiosis, antibiotics, probiotics, and fecal microbiota transplantation. *J Neurogastroenterol Motil* 2019;**25**:363–76. [PMC][[10.5056/jnm19044](http://dx.doi.org/10.5056/jnm19044)] [[31327219](http://www.ncbi.nlm.nih.gov/pubmed/31327219)][Mismatch] [InsertedFromOnline]

Gloor GB, Macklaim JM, Pawlowsky-Glahn V *et al.* Microbiome datasets are compositional: and this is not optional. *Front Microbiol* 2017;**8**:2224. [PMC][[10.3389/fmicb.2017.02224](http://dx.doi.org/10.3389/fmicb.2017.02224)] [[29187837](http://www.ncbi.nlm.nih.gov/pubmed/29187837)]

Gonzalez A, Navas-Molina JA, Kosciolek T *et al.* Qiita: rapid, web-enabled microbiome meta-analysis. *Nat Methods* 2018;**15**:796–8. [PMC][[10.1038/s41592-018-0141-9](http://dx.doi.org/10.1038/s41592-018-0141-9)] [[30275573](http://www.ncbi.nlm.nih.gov/pubmed/30275573)] [InsertedFromOnline]

Gonzalez CG, Mills RH, Zhu Q *et al.* Location-specific signatures of Crohn’s disease at a multi-omics scale. *Microbiome* 2022;**10**:133. [PMC][[10.1186/s40168-022-01331-x](http://dx.doi.org/10.1186/s40168-022-01331-x)] [[35999575](http://www.ncbi.nlm.nih.gov/pubmed/35999575)][Mismatch]

Gordon-Rodriguez E, Quinn T, Cunningham JP. Data augmentation for compositional data: advancing predictive models of the microbiome. *Adv Neural Inf Process Syst* 2022;**35**:20551–65.

He M, Zhao N, Satten GA. MIDASim: a fast and simple simulator for realistic microbiome data. *Microbiome* 2024;**12**:135. [PMC][[10.1186/s40168-024-01822-z](http://dx.doi.org/10.1186/s40168-024-01822-z)] [[39039570](http://www.ncbi.nlm.nih.gov/pubmed/39039570)]

Integrative HMP (iHMP) Research Network Consortium. The integrative human microbiome project: dynamic analysis of microbiome–host omics profiles during periods of human health and disease. *Cell Host Microbe* 2014;**16**:276–89.

Jiang Y, Atton M, Zhu Q *et al.* MIOSTONE: modeling microbiome-trait associations with taxonomy-adaptive neural networks. bioRxiv, 2023, preprint: not peer reviewed.[AQ18]

Knights D, Parfrey LW, Zaneveld J *et al.* Human-associated microbial signatures: examining their predictive value. *Cell Host Microbe* 2011;**10**:292–6. [PMC][[10.1016/j.chom.2011.09.003](http://dx.doi.org/10.1016/j.chom.2011.09.003)] [[22018228](http://www.ncbi.nlm.nih.gov/pubmed/22018228)]

Laske C, Müller S, Preische O *et al.* Signature of Alzheimer’s disease in intestinal microbiome: results from the AlzBiom study. *Front Neurosci* 2022;**16**:792996. [PMC][[10.3389/fnins.2022.792996](http://dx.doi.org/10.3389/fnins.2022.792996)] [[35516807](http://www.ncbi.nlm.nih.gov/pubmed/35516807)][Mismatch]

Lloyd-Price J, Arze C, Ananthakrishnan AN *et al.*, IBDMDB Investigators. Multi-omics of the gut microbial ecosystem in inflammatory bowel diseases. *Nature* 2019;**569**:655–62. [PMC][[10.1038/s41586-019-1237-9](http://dx.doi.org/10.1038/s41586-019-1237-9)] [[31142855](http://www.ncbi.nlm.nih.gov/pubmed/31142855)] [InsertedFromOnline]

Lozupone C, Knight R. UniFrac: a new phylogenetic method for comparing microbial communities. *Appl Environ Microbiol* 2005;**71**:8228–35. [PMC][[10.1128/AEM.71.12.8228-8235.2005](http://dx.doi.org/10.1128/AEM.71.12.8228-8235.2005)] [[16332807](http://www.ncbi.nlm.nih.gov/pubmed/16332807)]

McCoubrey LE, Elbadawi M, Orlu M *et al.* Harnessing machine learning for development of microbiome therapeutics. *Gut Microbes* 2021;**13**:1–20. [PMC][[10.1080/19490976.2021.1872323](http://dx.doi.org/10.1080/19490976.2021.1872323)] [[33522391](http://www.ncbi.nlm.nih.gov/pubmed/33522391)][Mismatch] [InsertedFromOnline]

Medina RH, Kutuzova S, Nielsen KN *et al.* Machine learning and deep learning applications in microbiome research. *ISME Commun* 2022;**2**:98. [PMC][[10.1038/s43705-022-00182-9](http://dx.doi.org/10.1038/s43705-022-00182-9)] [[37938690](http://www.ncbi.nlm.nih.gov/pubmed/37938690)]

Mills RH, Dulai PS, Vázquez-Baeza Y *et al.* Multi-omics analyses of the ulcerative colitis gut microbiome link bacteroides vulgatus proteases with disease severity. *Nat Microbiol* 2022;**7**:262–76. [PMC][[10.1038/s41564-021-01050-3](http://dx.doi.org/10.1038/s41564-021-01050-3)] [[35087228](http://www.ncbi.nlm.nih.gov/pubmed/35087228)] [InsertedFromOnline]

Mumuni A, Mumuni F. Data augmentation: a comprehensive survey of modern approaches. *Array* 2022;**16**:100258. [CrossRef][[10.1016/j.array.2022.100258](http://dx.doi.org/10.1016/j.array.2022.100258)]

Pedregosa F, Varoquaux G, Gramfort A *et al.* Scikit-learn: machine learning in Python. *J Mach Learn Res* 2011;**12**:2825–30.

Qin J, Li R, Raes J *et al.*; MetaHIT Consortium. A human gut microbial gene catalogue established by metagenomic sequencing. *Nature* 2010;**464**:59–65. [PMC][[10.1038/nature08821](http://dx.doi.org/10.1038/nature08821)] [[20203603](http://www.ncbi.nlm.nih.gov/pubmed/20203603)] [InsertedFromOnline]

Qin J, Li Y, Cai Z *et al.* A metagenome-wide association study of gut microbiota in type 2 diabetes. *Nature* 2012;**490**:55–60. [PMC][[10.1038/nature11450](http://dx.doi.org/10.1038/nature11450)] [[23023125](http://www.ncbi.nlm.nih.gov/pubmed/23023125)] [InsertedFromOnline]

Reiman D, Metwally AA, Sun J *et al.* PopPhy-CNN: a phylogenetic tree embedded architecture for convolutional neural networks to predict host phenotype from metagenomic data. *IEEE J Biomed Health Inform* 2020;**24**:2993–3001. [PMC][[10.1109/JBHI.2020.2993761](http://dx.doi.org/10.1109/JBHI.2020.2993761)] [[32396115](http://www.ncbi.nlm.nih.gov/pubmed/32396115)]

Rong R, Jiang S, Xu L *et al.* MB-GAN: microbiome simulation via generative adversarial network. *Gigascience* 2021;**10**:giab005. [PMC][[10.1093/gigascience/giab005](http://dx.doi.org/10.1093/gigascience/giab005)] [[33543271](http://www.ncbi.nlm.nih.gov/pubmed/33543271)][Mismatch]

Sayyari E, Kawas B, Mirarab S. TADA: phylogenetic augmentation of microbiome samples enhances phenotype classification. *Bioinformatics* 2019;**35**:i31–40. [PMC][[10.1093/bioinformatics/btz394](http://dx.doi.org/10.1093/bioinformatics/btz394)] [[31510701](http://www.ncbi.nlm.nih.gov/pubmed/31510701)]

Sekirov I, Russell SL, Antunes CM *et al.* Gut microbiota in health and disease. *Physiol Rev* 2010;**90**:859–904. [PMC][[10.1152/physrev.00045.2009](http://dx.doi.org/10.1152/physrev.00045.2009)] [[20664075](http://www.ncbi.nlm.nih.gov/pubmed/20664075)] [InsertedFromOnline]

Sharma D, Paterson AD, Xu W. TaxoNN: ensemble of neural networks on stratified microbiome data for disease prediction. *Bioinformatics* 2020;**36**:4544–50. [PMC][[10.1093/bioinformatics/btaa542](http://dx.doi.org/10.1093/bioinformatics/btaa542)] [[32449747](http://www.ncbi.nlm.nih.gov/pubmed/32449747)]

Sharma D, Lou W, Xu W. phylaGAN: data augmentation through conditional GANs and autoencoders for improving disease prediction accuracy using microbiome data. *Bioinformatics* 2024;**40**:btae161. [PMC][[10.1093/bioinformatics/btae161](http://dx.doi.org/10.1093/bioinformatics/btae161)] [[38569898](http://www.ncbi.nlm.nih.gov/pubmed/38569898)][Mismatch]

Turnbaugh PJ, Ley RE, Mahowald MA *et al.* An obesity-associated gut microbiome with increased capacity for energy harvest. *Nature* 2006;**444**:1027–31. [PMC][[10.1038/nature05414](http://dx.doi.org/10.1038/nature05414)] [[17183312](http://www.ncbi.nlm.nih.gov/pubmed/17183312)]

Vogt NM, Kerby RL, Dill-McFarland KA *et al.* Gut microbiome alterations in Alzheimer’s disease. *Sci Rep* 2017;**7**:13537. [PMC][[10.1038/s41598-017-13601-y](http://dx.doi.org/10.1038/s41598-017-13601-y)] [[29051531](http://www.ncbi.nlm.nih.gov/pubmed/29051531)][Mismatch] [InsertedFromOnline]

Washburne AD, Morton JT, Sanders J *et al.* Methods for phylogenetic analysis of microbiome data. *Nat Microbiol* 2018;**3**:652–61. [PMC][[10.1038/s41564-018-0156-0](http://dx.doi.org/10.1038/s41564-018-0156-0)] [[29795540](http://www.ncbi.nlm.nih.gov/pubmed/29795540)]

Wu S, Chen Y, Li Z *et al.* Towards multi-label classification: next step of machine learning for microbiome research. *Comput Struct Biotechnol J* 2021;**19**:2742–9. [PMC][[10.1016/j.csbj.2021.04.054](http://dx.doi.org/10.1016/j.csbj.2021.04.054)] [[34093989](http://www.ncbi.nlm.nih.gov/pubmed/34093989)]

Xiao J, Chen L, Johnson S *et al.* Predictive modeling of microbiome data using a phylogeny-regularized generalized linear mixed model. *Front Microbiol* 2018;**9**:1391. [PMC][[10.3389/fmicb.2018.01391](http://dx.doi.org/10.3389/fmicb.2018.01391)] [[29997602](http://www.ncbi.nlm.nih.gov/pubmed/29997602)]

Zhang C-B, Jiang P-T, Hou Q *et al.* Delving deep into label smoothing. *IEEE Trans Image Process* 2021;**30**:5984–96. [CrossRef][[10.1109/TIP.2021.3089942](http://dx.doi.org/10.1109/TIP.2021.3089942)]

Zhang H, Cisse M, Dauphin YN *et al.* mixup: Beyond empirical risk minimization. In: *International Conference on Learning Representations*. 2018.[AQ19]

Zhang L, Yang Z, Yang D. TreeMix: compositional constituency-based data augmentation for natural language understandingIn: *Conference of the North American Chapter of the Association for Computational Linguistics (NAACL)*. 2022.[AQ20]

Zhu Q, Mai U, Pfeiffer W *et al.* Phylogenomics of 10,575 genomes reveals evolutionary proximity between domains bacteria and archaea. *Nat Commun* 2019;**10**:5477. [PMC][[10.1038/s41467-019-13443-4](http://dx.doi.org/10.1038/s41467-019-13443-4)] [[31792218](http://www.ncbi.nlm.nih.gov/pubmed/31792218)] [InsertedFromOnline]

Zhu Q, Hou Q, Huang S *et al.* Compositional and genetic alterations in Graves’ disease gut microbiome reveal specific diagnostic biomarkers. *ISME J* 2021;**15**:3399–411. [PMC][[10.1038/s41396-021-01016-7](http://dx.doi.org/10.1038/s41396-021-01016-7)] [[34079079](http://www.ncbi.nlm.nih.gov/pubmed/34079079)][Mismatch] [InsertedFromOnline]

Zhu Q, Huang S, Gonzalez A *et al.* Phylogeny-aware analysis of metagenome community ecology based on matched reference genomes while bypassing taxonomy. *mSystems* 2022;**7**:e00167–22. [PMC][[10.1128/msystems.00167-22](http://dx.doi.org/10.1128/msystems.00167-22)] [[35369727](http://www.ncbi.nlm.nih.gov/pubmed/35369727)][Mismatch] [InsertedFromOnline]

Figure 1. Overview of PhyloMix. (a) PhyloMix generates synthetic samples with artificial labels based on existing data and incorporates them into the training data to enhance the performance of general ML models. (b) PhyloMix leverages phylogeny to generate synthetic samples by removing a subtree from one sample and recombining it with the counterpart from another sample. The involved subtree, along with the affected probabilistic phylogenetic profile, is highlighted in orange.[AQ21]

Figure 2. Data augmentation performance in the supervised learning setting. (a) The evaluation was performed on real microbiome datasets with varying microbial taxa sizes. PhyloMix is evaluated alongside five ML models with varying predictive capabilities and compared against four distinct baseline methods. The augmentation generated by PhyloMix preserves or notably enhances predictive performance, regardless of the dataset complexity or the sophistication of the ML models used. For scientific rigor, the performance comparison between PhyloMix and other baseline methods is quantified using one-tailed two-sample *t*-tests to calculate *P*-values: *****P*-value ≤ 0.0001; ****P*-value ≤ 0.001; ***P*-value ≤ 0.01; **P*-value ≤ 0.05; ns: *P*-value > 0.05. (b) PhyloMix is compared to baseline methods using radar plots, with performance evaluated based on relative AUPRC, normalized against the best-performing method across all methods. Each dot corresponds to the performance with or without data augmentation for a given method under a random seed.

Figure 3. Data augmentation performance in the representation learning setting. The evaluation was conducted on six publicly available microbiome datasets and compared against six distinct baseline methods. The representations obtained through PhyloMix consistently outperform those generated by other baseline methods in predicting disease status, measured by both AUPRC and AUROC. The performance comparison between PhyloMix and other baseline methods is quantified using one-tailed two-sample *t*-tests to calculate *P*-values.

Figure 4. Dissecting the performance of PhyloMix through control studies. (a) The data augmentation by PhyloMix incurs only moderate computational overhead, consisting of two main components: the generation of synthetic samples and the additional training time required to incorporate these synthetic samples. (b) The choice of the Beta distribution is crucial in determining the sample mixing weights. In general, this choice does not negatively impact data augmentation performance. The only exception is the logistic regression model, where data augmentation transforms logistic regression into linear regression, leading to a performance downgrade if the Beta distribution is not chosen properly. (c) PhyloMix is robust to the number of augmented samples. Data augmentation consistently improves predictive performance. However, increasing the number of augmented samples inevitably leads to higher computational overhead. (d) PhyloMix demonstrates more significant improvements when the training data size is small. (e) PhyloMix smooths the decision boundary of the SVM model on the IBD dataset.

AQ1: The running head has been inserted. Please edit, if necessary, by making changes directly in the text.

AQ2: Please check the spelling and accuracy of all author names and affiliations, particularly for any co-authors. Also check that author surnames are correctly highlighted. This is to ensure that forenames and surnames are tagged correctly for online indexing. Incorrect names and affiliations may lead to an author not being credited for their work by funders, institutions, or other third parties. Make any changes directly in the text. Note that changes to your authorship list (adding or removing authors, changing designation of corresponding author) require additional approvals. Email jnls.author.support@oup.com if this is required.

AQ3: Please provide Postal code/Zip code for affiliation 1 directly in the text.

AQ4: Please provide Postal code/Zip code for affiliation 2 directly in the text.

AQ5: Please note that we have multiple versions of the corresponding author and the email address for the corresponding author. Check the version used here and make any changes directly in the text.

AQ6: Please provide the complete details (department/unit, university/institution, and postal address with road/street name) for the corresponding author directly in the text.

AQ7: Per journal style, heading levels appear in a specific order: Introduction, Materials and methods, Results, Discussion, and Conclusion. Please check and make changes directly in the text.

AQ8: If your manuscript has figures or text from other sources, please ensure you have permission from the copyright holder. Also ensure that the copyright owner is properly credited, for instance in the figure legends. Make any changes directly in the text. For any questions about permissions, contact jnls.author.support@oup.com.

AQ9: Plase check and confirm the citation for sections throughout and correct directly in the text.

AQ10: We have received the following files for publication as supplementary material.

- phylomix_supp

These will be published in the format provided, without edits. If any files need to be removed from this list, specify by responding to this query, stating the filename. If any files need to be added, upload them directly or email them with your proof corrections. If any files need to be updated, upload them directly or email them, without altering the filename. We will seek editor approval for any changes to supplementary material, which may delay publication of your article.

AQ11: Please ensure all "conflicts of interest" (Or "disclosures") have been included for you and your co-authors, and that this section is correct. Edit the text directly if changes are required.

AQ12: Please check that funding is recorded in a separate funding section, if applicable. Use the full official names of any funding bodies and include any grant numbers. Insert any changes to the funding section directly in the text.

AQ13: Journal policy requires authors to provide a data availability statement in their manuscript. Please confirm that this statement is included in your manuscript and that any required links or identifiers for your data are present in the manuscript as described or provide edits with the required information directly in the text. See the sample data availability statements at https://academic.oup.com/journals/pages/authors/preparing_your_manuscript/research-data-policy#data2

AQ14: Please provide the page range for reference Adebayo et al. (2018) directly in the reference.

AQ15: Please provide updated publication details for preprint reference Cao et al. (2020), if available, directly in the reference.

AQ16: Please provide the journal title, volume number for reference Chen et al. (2020) directly in the reference.

AQ17: Please provide the publisher location (city/state/country), publisher name and location where the proceedings were held for reference Davis and Goadrich (2006) directly in the reference.

AQ18: Please provide updated publication details and doi for preprint reference Jiang et al. (2023), if available, directly in the reference.

AQ19: Please provide the publisher location (city/state/country), publisher name and location where the proceedings were held for reference Zhang et al. (2018) directly in the reference.

AQ20: Please provide the publisher location (city/state/country), publisher name and location where the proceedings were held for reference Zhang et al. (2022) directly in the reference.

AQ21: The caption for Figure 1 currently contains reference to colour used within the figure. For accessibility purposes reference to colour should be avoided. If possible, please update the wording for the figure caption to remove reference to colour.
